# Glycan Markers of Human Stem Cells Assigned with Beam Search Arrays*[Fn FN2]

**DOI:** 10.1074/mcp.RA119.001309

**Published:** 2019-07-15

**Authors:** Nian Wu, Lisete M. Silva, Yan Liu, Yibing Zhang, Chao Gao, Fuming Zhang, Li Fu, Yanfei Peng, Robert Linhardt, Toshisuke Kawasaki, Barbara Mulloy, Wengang Chai, Ten Feizi

**Affiliations:** ‡ Glycosciences Laboratory, Department of Medicine, Imperial College London, London W12 0NN, United Kingdom; § Department of Chemical and Biological Engineering, Center for Biotechnology and Interdisciplinary Studies, Rensselaer Polytechnic Institute, Troy, New York 12180; ¶ Research Center for Glycobiotechnology, Ritsumeikan University, Noji-Higashi, 1–1-1, Kusatsu Shiga 525–8577, Japan; ‖ Department of Surgery, Beth Israel Deaconess Medical Center, Harvard Medical School, Boston, Massachusetts 02215

**Keywords:** Stem cells, glycomics, micro arrays, mass spectrometry, NMR, growth factors, antibodies, glycan arrays, glycan markers, glycan roles, keratan sulfate

## Abstract

Glycan antigens recognized by monoclonal antibodies have served as stem cell markers. To understand regulation of their biosynthesis and their roles in stem cell behavior precise assignments are required. We have applied state-of-the-art glycan array technologies to compare the glycans bound by five antibodies that recognize carbohydrates on human stem cells. These are: FC10.2, TRA-1–60, TRA-1–81, anti-i and R-10G. Microarray analyses with a panel of sequence-defined glycans corroborate that FC10.2, TRA-1–60, TRA-1–81 recognize the type 1-(Galβ-3GlcNAc)-terminating backbone sequence, Galβ-3GlcNAcβ-3Galβ-4GlcNAcβ-3Galβ-4GlcNAc, and anti-i, the type 2-(Galβ-4GlcNAc) analog, Galβ-4GlcNAcβ-3Galβ-4GlcNAcβ-3Galβ-4GlcNAc, and we determine substituents they can accommodate. They differ from R-10G, which requires sulfate. By *Beam Search* approach, starting with an antigen-positive keratan sulfate polysaccharide, followed by targeted iterative microarray analyses of glycan populations released with keratanases and mass spectrometric monitoring, R-10G is assigned as a mono-sulfated type 2 chain with 6-sulfation at the penultimate *N*-acetylglucosamine, Galβ-4GlcNAc(6S)β-3Galβ-4GlcNAcβ-3Galβ-4GlcNAc. Microarray analyses using newly synthesized glycans corroborate the assignment of this unique determinant raising questions regarding involvement as a ligand in the stem cell niche.

## Abstract (precis)

R-10G antigen of human iPS and ES cells has been assigned as a unique mono-sulfated glycan. We highlight its relationship to a known bioactive glycan sequence, the ligand on high endothelial venules for the lymphocyte homing receptor, L-selectin. Details of the sequences of four other glycan antigens on the podocalyxin are re-evaluated and ambiguities resolved. Regulation of biosynthesis and possible involvements of these glycans in podocalyxin-signaling in stem cells are discussed.

## Graphical Abstract

**Figure:**
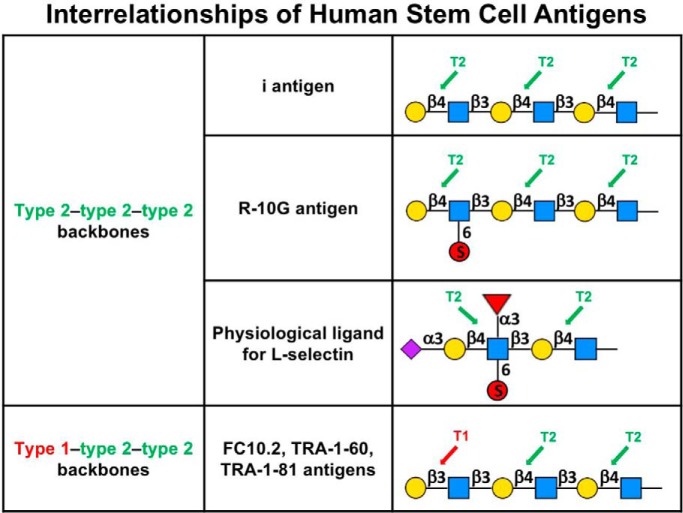

**Highlights**

- R-10G is a mono-sulfated glycan antigen of human iPS and ES cells on podocalyxin.
- R-10G and nonsulfated i are reciprocal antigens on type 2 glycan backbones.
- TRA-1–60 and -81 and FC10.2 are antigens expressed on unsulfated type 1 backbones.
- These assignments open the way to probing their roles in podocalyxin-signaling.

Cellular glycans were historically among the earliest biochemical and immunochemical markers in embryonic developments (1–3). Using as reagents glycan sequence-specific monoclonal antibodies (initially, natural human antibodies followed by murine hybridoma-derived) remarkable insights were gained into the expression and polarization of glycans in post-implantation embryos and teratocarcinomas of mouse associated with cell differentiation (4, 5). We overview here below the glycan antigens on murine and human stem cells that have been reported over the years. These antigens have been used as markers of stem cells (1) although little is known about their roles in stem cell behavior.

Undifferentiated murine embryonal carcinoma cells were observed to be rich in surface-associated and cytoplasmic I antigen (4). This antigen is recognized by human monoclonal anti-I Ma and anti-I Step; these being designations of monoclonal antibodies in sera of cases of cold agglutinin disease (6). These antibodies are directed at different domains on the branched structures of the poly-*N*-acetyllactosamine series as indicated in structures 1 and 2 (Table I). These are glycan sequences consisting of repeated type 2 (T2, Galβ-4GlcNAc) on glycoproteins and glycolipids, where Gal is galactose and GlcNAc is *N*-acetylglucosamine. Differentiation into primary endoderm was observed to be associated with the appearance of i antigen (4) recognized by the human anti-i Den, which is directed at the unbranched repeated T2 sequence as in structure 3 (Table I). With the advent of hybridoma technology, there followed the demonstration that the 8-cell stage-specific embryonic antigen of mouse recognized by mAb anti-SSEA-1 is expressed on the α-3 fucosylated forms of the I and i antigens (termed Lewis^X^), structures 4 and 5, respectively (5) (Table I). These antigens were detected predominantly on high molecular weight glycoproteins (7, 8) the common feature being the abundance of repeated T2 backbone sequences.

**Table I:**
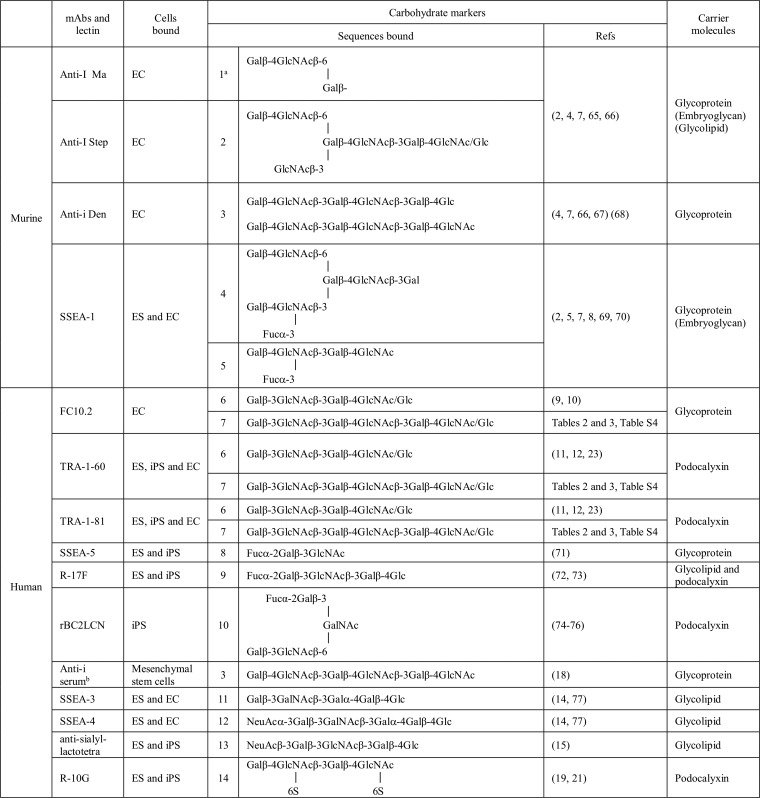
Murine and human stem cell-associated carbohydrate markers and carrier molecules

^a^ Numbers in columns 4 are designations of the sequences cited in the text. Glycan sequences recognized by anti-I Ma and Step, anti-i Den, mAbs FC10.2, TRA-1–60 and TRA-1–81 were originally determined using glycolipids or glycans with Glc at the reducing ends. On stem cells they occur predominantly on glycoproteins.

^b^ An unnamed human serum.

In contrast with the murine embryonic endoderm and embryonal carcinoma-associated antigens, the human embryonic endoderm- and embryonal carcinoma-associated antigen recognized by the monoclonal mAb FC10.2 was assigned as the lacto-*N*-tetraose sequence, with T1 (Galβ-3GlcNAc) -T2 backbone sequence, structure 6 (Table I); this was detected on a glycoprotein with an apparent molecular weight, ∼200 kDa, (9, 10) similar to that of podocalyxin (11). This glycoprotein is the carrier of the antigen(s) recognized by more recently generated mAbs, TRA-1–60 and TRA-1–81, which recognize human stem cells (12), and, like FC10.2, bind to the T1-T2 sequence, structures 6 and 7 (Table I). The expression of this antigenic activity is reported to be common to various types of human stem cells: embryonic stem cell (ES)^1^, induced pluripotent stem cell (iPS), and embryonal carcinoma-associated stem cell (EC) (12, 13).

Three other T1-related antigens common to human ES and iPS have been identified: two are T1-based blood group H recognized by mAbs anti-SSEA-5 and R-17F, structures 8 and 9 (Table I). The third is branched sequence structure 10 (Table I), containing blood group H based on the T3 (Galβ-3GalNAc) backbone (where GalNAc is *N*-acetylgalactosamine). This antigen is bound by the lectin rBC2LCN, and reported to be cross-reactive with T1-based blood group H, and expressed on podocalyxin. The R-17F antigen is additionally borne on a glycolipid. Three other glycolipid-borne antigens on human ES and EC recognized by mAbs anti-SSEA-3, anti-SSEA-4 and anti-Sialyl lactotetra antibodies are structures 11, 12 and 13 respectively (Table I), of which the first two are of the globo series based on Galβ-3GalNAcβ-3Galα-4Galβ-4Glc (14) and the third is based on the type 1 lacto series, Galβ-3GlcNAcβ-3Galβ-4Glc (15). Other studies have shown that not only the terminal epitopes but also the core structures are differentially expressed in stem *versus* differentiated cells (16, 17).

T2-based human stem cell antigens have been two in number. The first is a human mesenchymal stem cell-associated antigen expressed on the repeated T2-T2 based backbone, namely, i antigen (18). The second is recognized by mAb R-10G, which distinguishes human ES and iPS from human EC cells (19). It was shown that binding of mAb R-10G to an antigen-positive glycoprotein was unaffected by its treatment with neuraminidases and fucosidases. The antibody was shown to bind to the polysaccharide keratan sulfate (KS), which is based on long repeated T2-T2 sequences (19). A highly sulfated KS from bovine articular cartilage (with sulfation on many of the GlcNAc and Gal residues) was lacking in R-10G antigen activity, whereas bovine corneal KS, which contains some regions with sulfated GlcNAc and nonsulfated Gal residues (20), was antigen-positive (19). For these reasons, the R-10G antigen was deduced to be expressed on an under-sulfated form of KS. While the present study was under way a KS-related tetrasaccharide, structure 14, which has sulfate at position 6 of each of two GlcNAc residues (Table I) was chemically synthesized and when biotinylated was bound by mAb R-10G (21).

Knowledge of the glycan markers has not yet been matched by knowledge of the regulation of their biosynthesis and their functions in the stem cell niche. There also remain some ambiguities in reports of the binding specificities, for example those of the TRA-1–60 and -81 toward KS-like saccharides (19, 22, 23). Glycan arrays, since their inception (24) have been a powerful means of analyzing glycan-binding specificities of diverse carbohydrate-recognition systems (25, 26). The continued expansion of libraries of sequence-defined glycan probes offers increasing opportunities to keep under review the repertoires of glycans recognized by the anti-stem cell antibodies. There are moreover technological advances that enable the generation of “bespoke” glycome probes, which we have termed *Designer* arrays (27) and Beam Search arrays (28) from antigen positive macromolecules to detect, isolate and characterize natural determinants.

Here we use an array of sequence-defined glycans to make for the first time a close comparison of the glycan-binding specificities of the five antibodies that recognize human stem cells: mAbs TRA-1–60, TRA-1–81 and FC10.2 that broadly recognize ES, iPS and EC cells, an anti-i P1A ELL directed at linear poly-*N*-acetyllactosamine sequences (29), which according to ref (30) is predicted to recognize mesenchymal stem cells, and R-10G that recognizes ES and iPS cells. To assign the determinant of mAb R-10G, which is clearly different from those of the four other antibodies, we use the Beam Search microarray approach (28) using an antigen-positive KS polysaccharide as the macromolecular start-point. This is followed by a targeted, iterative microarray analyses and concomitant mass spectrometry of glycan populations derived from the polysaccharide after partial depolymerization by keratanase I or keratanase II or acid hydrolysis. Microarray analyses with newly synthesized glycans corroborate the assignment we have made for the unique sequence of R-10G antigen associated with ES and iPS cells.

## EXPERIMENTAL PROCEDURES

The glycan array-based data are reported in accordance with MIRAGE (Minimum Information Required for A Glycomics Experiment) Glycan Microarray Guidelines (31). These include details of the glycan binding samples investigated (antibodies, growth factors and morphogens) and their detection systems, the saccharides and the neoglycolipids (NGLs) in the glycan libraries for different array sets, the conditions used for array construction, as well as information on image and data analyses: these are summarized in the Supplemental Glycan Microarray document based on MIRAGE Guidelines (supplemental Table S2). The experimental data not covered are given below.

### 

#### 

##### Keratanases

Keratanase I from *Pseudomonas sp*. (EC 3.2.1.103) was from Seikagaku (Joetsu, Japan). Recombinant Keratanase II from *Bacillus circulans.* (EC 3.2.1) was as prepared according to (32). Please note that to avoid confusion in the names of the two KS specific enzymes, the term “keratanase I” was used throughout the paper instead of the commonly used “keratanase.”

##### Partial Depolymerization of KS and Fractionation of KS Derived Glycans

The conditions for partial digestion of KS with keratanase I were adapted from those described by Nakazawa and Suzuki (33). In brief, KS (10 mg) was incubated with 0.5 unit of keratanase I in 0.5 ml Tris-HCl buffer (20 mm, pH 7.4) at 37 °C. Partial digestion of KS with keratanase II was performed as described by Brown *et al.* (34). In brief, KS (20 mg) was incubated with 0.96 unit of keratanase II in 2 ml NH_4_OAc buffer (50 mm, pH 7.4) at 37 °C. For partial acid hydrolysis, KS (1.4 mg) was incubated with 0.1 m HCl at 80 °C. The partial depolymerization of bovine corneal KS using keratanase I, keratanase II and acid hydrolysis was monitored by Superdex Peptide column (1 × 30 cm, GE Healthcare, Fairfield, Connecticut) and ESI-MS, and stopped at 23 h, 7.5 h and 4 h, respectively, at the disappearance of the peak at void volume (supplemental Fig. S1). The keratanase digestion reactions were stopped by heating the solutions in a water bath at 100 °C for 1 min, whereas the acid hydrolysis was stopped at the indicated times by neutralizing with 0.1 m NaOH. The reaction mixtures were lyophilized.

##### Preparation of Fractions of KS Glycome

Bio-Gel P-6 (1.6 × 90 cm, Bio-Rad Laboratories, Hercules, California) was used for size fractionation of glycans after keratanase treatment of KS, and elution was with 200 mm NH_4_Cl at a flow rate of 15 ml/h. Eluates were monitored by a refractive index (RI) detector, fractions were pooled as indicated (supplemental Fig. S2*A* and S2*B*) and lyophilized. Sephadex G10 column (1.6 × 35 cm, GE Healthcare) was used for desalting of fractions collected from Bio-Gel P-6 chromatography. Deionized water was used for elution at a flow rate of 20 ml/h and eluates were monitored with a RI detector. Superdex Peptide column (GE Healthcare, 1 × 30 cm) was used to fractionate the products obtained after acid hydrolysis of KS. Elution was with 50 mm NH_4_OAc at a flow rate of 0.3 ml/min and monitored by RI. Fractions were pooled as indicated and lyophilized and were coevaporated repeatedly with Deionized water by freeze-drying to remove the volatile buffer salt NH_4_OAc (supplemental Fig. S2*C*). Selected fractions were analyzed by ESI-MS to determine chain lengths and sulfate contents (supplemental Table S6). The molar quantities of the glycans in the KS fractions were estimated based on the galactose content and molecular mass determined by MS analysis. The galactose contents were determined by established dot orcinol-sulfuric acid method on TLC plates, using galactose as standard (35).

Strong anion-exchange (SAX) column (4.6 × 250 mm, Waters, Milford, Massachusetts) or HiTrap ion exchange cartridge (7 × 25 mm, GE Healthcare) or hypercarb porous graphitized carbon (PGC) column (30 × 4.6 mm, Thermo Scientific, Waltham, Massachusetts) were used for fractionation of glycans by HPLC (Gilson, Madison, Wisconsin). Elution was carried out by a gradient of LiClO_4_ for both SAX-HPLC and HiTrap cartridge (Solvent 1: 10 mm LiClO_4_ and Solvent 2: 500 mm LiClO_4_). Chromatographies by SAX and HiTrap were performed in 40 min at a flow rate of 1 ml/min with detection by UV at 206 nm. For PGC-HPLC the elution was by a gradient of H_2_O/ACN (Solvent 1: H_2_O; Solvent 2: ACN/H_2_O: 80:20; both containing 0.05% trifluoroacetic acid) in 40 min at a flow rate of 0.5 ml/min with detection by UV at 206 nm. The gradients used are given in the figures. Fractions were pooled as indicated in the respective figures. The fractions obtained from SAX-HPLC and HiTrap were lyophilized and desalted with a Superdex Peptide column and lyophilized as described above. To fractions obtained from PGC-HPLC were added 50 mm NH_4_HCO_3_ before removal of the ACN under N_2_ stream and the TFA by repeated lyophilization.

##### Desulfation of KS Glycans

Desulfation of KS glycans was performed as described (36). After several preliminary experiments, the conditions selected were as follows: freeze-dried hexasaccharides substituted with 4 to 6 sulfates (∼100 nmol) were added to 150 μl of 20 mm methanolic HCl for incubation at room temperature for 17 h. The reactions were stopped by immediate removal of the methanolic HCl reagent after addition of 50 μl of 50 mm NH_4_HCO_3_. The reaction product was dried by lyophilization.

##### Lipid Conjugation of KS Oligosaccharides to Prepare NGLs

NGLs were prepared from reducing KS glycans by oxime-ligation using the lipid reagent *N*-aminooxyacetyl-1,2-dihexadecyl-*sn*-glycero-3-phosphoethanolamine, AOPE, essentially as described (37). The reactions were on a scale between 10 nmol and 100 nmol of starting oligosaccharides depending on their availability. In brief, for short chain glycans (di- to octa-saccharides), five molar equivalents of AOPE solution (10 nmol/μl) were added, whereas 10 molar equivalents were added to longer glycans. The incubations were at 37 °C for 16 h (shorter glycans) or 32 h (longer glycans). The purification and quantitation of NGLs were as described (35).

##### MS Analysis

Negative ion ESI-MS and CID-MS/MS were carried out on a Waters (Manchester, UK) Q-TOF-type mass spectrometer SYNAPT-G2. Cone voltage was at 80 eV and capillary voltage at 3 kV. Source temperature was at 80 °C and the desolvation temperature at 150 °C. The resolution was set at 20,000 for MS and 8,000 for CID-MS/MS with a scan rate at 1.5 s for the mass range 100–2000. Product-ion spectra were obtained using argon as the collision gas at a pressure of 1.7 bar. The collision energy was adjusted between 12 and 20 eV for optimal fragmentation. A scan rate of 1.5 s/scan was used for both ESI-MS and MS/MS experiments and the acquired spectra were summed for presentation. For analysis, glycans were dissolved in water and NGLs in CHCl_3_/MeOH/H_2_O, 25:25:8 (v/v/v) typically at a concentration of 10–20 pmol/μl, of which 1 μl was injected via a HPLC injector. Solvent (ACN/2 mm NH_4_HCO_3_, 1:1, or CHCl_3_/MeOH/H_2_O, 25:25:8) was delivered by a HPLC pump (Waters) at a flow rate of 10 μl/min.

##### NMR Spectroscopy

^1^H-NMR, as well as 2D ^13^C-^1^H HSQC and TOCSY NMR spectra of glycans were recorded at 700 MHz using a BruckerAvance spectrometer (Karlsruhe, Germany) equipped with cryoprobe, in D_2_O solution at 30 °C. 1D and 2D pulse sequences were supplied by the equipment manufacturer. The observed ^1^H chemical shifts were relative to internal acetone (2.225 ppm). Each sample (∼240 μg) was taken up in D_2_O 99.9% (Apollo Scientific, Stockport, UK) exchanged by lyophilization, then re-dissolved in 300 μl D_2_O and transferred to a Shigemi NMR microtube (Sigma, St. Louis, Missouri).

##### Generation of Microarrays of Polysaccharides and NGLs and Microarray Analysis

The generation of microarrays of polysaccharides and NGLs is essentially as described (38). There were six microarrays: one of glycosaminoglycan (GAG) polysaccharides and five of lipid-linked glycans collectively termed NGL arrays, of which one contained 79 sequence-defined NGL probes, in house designated CLL microarray, and four NGL Beam Search arrays. The details are summarized in supplemental Table S2.

Microarray analyses were performed essentially as described (38) at ambient temperature except for anti-i P1A ELL at 4 °C. Concentrations of the antibodies analyzed, the blockers and diluents, as well as the concentrations of polysaccharides and lipid-linked probes arrayed are as described in supplemental Table S2.

R-10G was analyzed at 100 μg/ml without precomplexation in the analyses with CLL microarray and KS-derived Beam Search arrays, and at 10 μg/ml with precomplexation in analyses with polysaccharide microarray. For precomplexation, R-10G was incubated for 30 min with the goat anti-mouse IgG (detection antibody) at a ratio of 1:3 (w/w) before overlay for 90 min.

The other antibodies MZ15, 5D4, CS56, anti-i P1A ELL, FC10.2, TRA-1–60, TRA-1–81 and anti-LNT were analyzed in CLL microarray, polysaccharide microarray and KS-derived Beam Search arrays, without precomplexation as indicated (supplemental Table S2).

Assay conditions used for the exploratory microarray analyses of recombinant proteins FGF1, FGF2, Shh, Wnt-3a, BMP-2 and BMP-4 are described in supplemental Table S2.

## RESULTS

### 

#### 

##### A Close Comparison of Glycans Recognized by mAbs R-10G, anti-i P1A ELL, FC10.2, TRA-1–60 and TRA-1–81 with Sequence-defined Glycans Based on T1-T2 and T2-T2 Backbones

Initial analyses were performed with R-10G, anti-i P1A ELL, FC10.2, TRA-1–60, and TRA-1–81 using a microarray of sequence-defined glycans based on T1-T2 and T2-T2 backbones (supplemental Table S3). This was (a) to determine any binding of R-10G to T2-T2 backbones of the type that occur in KS, but without sulfation, compared with anti-i P1A ELL, and (b) to make the close comparison hitherto not possible, between TRA-1–60 and TRA-1–81 with FC10.2 with respect to glycan sequences they recognize. For reference, anti-LNT directed at the T1-T2 backbones was also analyzed.

R-10G gave no significant binding to the glycans arrayed (Table II and supplemental Table S4). This contrasted with the strong binding observed with anti-i P1A ELL to the nonsubstituted T2-T2 structure at position 14 and the 6-sialylated structure at positions 16 and 17 in the array (supplemental Table S4).

**Table II:**
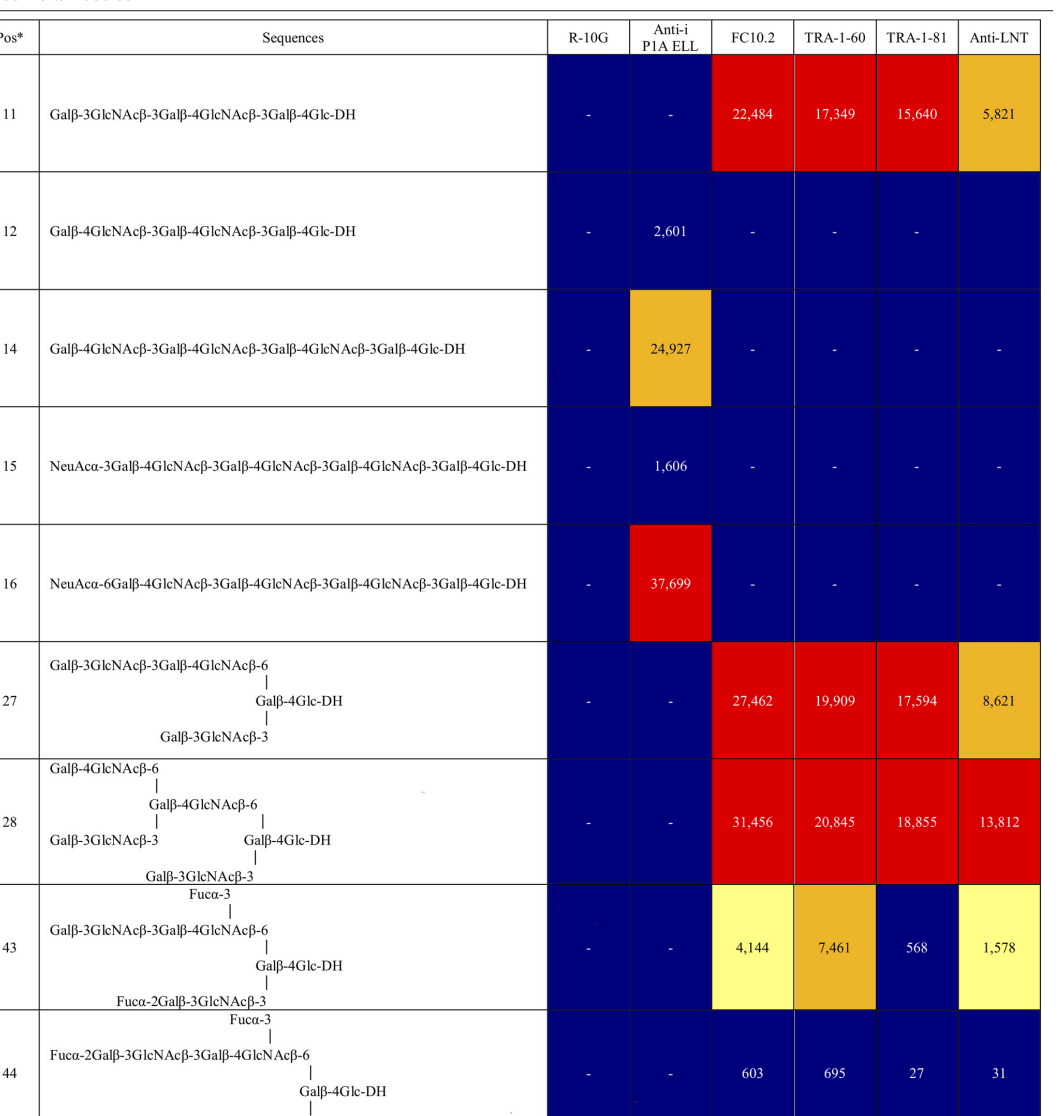
Relative intensities of binding of mAbs R-10G, anti-i P1A ELL, FC10.2, TRA-1–60, TRA-1–81, and anti-LNT to antigen-positive and related antigen-negative probes in sequence-defined NGL microarray (in house designation “CLL microarray set”) Full data are in supplemental Table S4. Binding scores are means of the fluorescence intensities at 5 fmol/probe spot; - indicates the score is less than 1. Relative binding intensity: 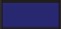
< 10%, 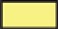
10–30%, 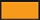
30–70%, 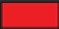
70–100%, where 100% is the maximum score for respective antibodies.

^a^ Pos, position in the microarray.

FC10.2, TRA-1–60, TRA-1–81 gave strong binding signals with three structures at positions 11, 27 and 28 in the array (Table II and supplemental Table S4). The common feature is the presence of nonreducing end T1 followed by internal T2 as a part of linear or branched glycan chains. In this respect, they resembled the commercial anti-LNT (supplemental Table S4). Although the glycan probes had glucose (Glc) at the reducing end, there would be GlcNAc here as part of the *O*-glycan sequences of a glycoprotein such as podocalyxin (39). Differences were revealed among these four antibodies: FC10.2, anti-LNT and TRA-1–60 but not TRA-1–81 gave binding albeit weak to the structure with a fucose (Fuc) 3-linked to the inner GlcNAc (position 43), and the binding was abolished in the presence of 2-linked Fuc at the nonreducing end (position 44).

In sum, the three anti-stem cell mAbs FC10.2, TRA-1–60 and TRA-1–81 are very similar in their strong binding to the nonsubstituted T1-T2 sequences as part of linear or branched glycans longer than tetrasaccharide. Unlike anti-i P1A ELL, R-10G gave no detectable binding to nonsulfated T2-T2 sequences.

##### Corneal KS Selected for Beam Search Array

**Fig. 1.**
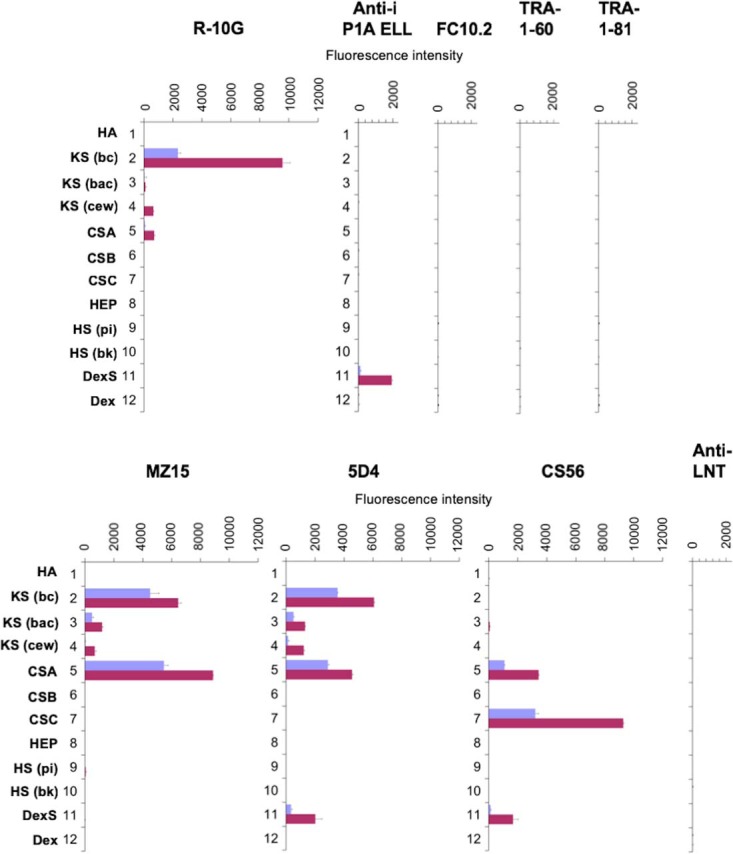
**Screening analyses of anti-stem cell and reference antibodies using the GAG polysaccharide microarray.** The four stem cell antibodies, R-10G, FC10.2, TRA-1–60 and TRA-1–81 were analyzed together with mAbs MZ15, 5D4, CS56, anti-i P1A ELL and anti-LNT as controls. Abbreviations for the polysaccharides: hyaluronic acid (HA); keratan sulfate (KS) isolated from bovine cornea (bc), bovine articular cartilage (bac) and chicken egg white (cew); chondroitin sulfate (CS) A, B and C; heparin, heparan sulfate (HS) isolated from porcine intestinal mucosa (pi) and bovine kidney (bk); Dextran sulfate (DexS); Dextran T2000 (Dex). Detection of binding was as described (supplemental Table S2). Binding scores for duplicate spots at 90 and 300 pg per spot are in blue and red respectively, error bars represent half of the difference between the two scores. The unpredicted binding by the anti-KS to the commercial CSA is attributed to the variable presence of KS antigen in different batches as depicted in supplemental Table S1.

R-10G, anti-i P1A ELL, FC10.2, TRA-1–60 and TRA-1–81 were next analyzed using the GAG polysaccharide microarray (Fig. 1). The array included three KS preparations isolated from bovine cornea, bovine articular cartilage and chicken egg white, and commercial preparations of chondroitin sulfates (CS) A, B, and C, heparin, two heparan sulfates isolated from porcine intestinal mucosa and bovine kidney, and hyaluronan. For reference, dextran and highly sulfated dextran were also analyzed.

Among the five anti-stem cell mAbs, R-10G gave strong binding only to the corneal KS, very weak binding to the egg white KS and negligible binding with the articular cartilage KS. There was no significant binding observed to any of the other types of GAGs except very weak binding to CSA (Fig. 1). With the anti-i P1A ELL serum the only significant binding detected was to the dextran sulfate (Fig. 1). No binding was detected with FC10.2, TRA-1–60, and TRA-1–81.

Among the antibodies included for reference, the anti-KS mAbs MZ15 and 5D4 bound strongly to corneal KS, as predicted. They also bound to the two other KS preparations (Fig. 1). CS56 gave binding signals with the Sigma CSA and CSC in accordance with previous knowledge (40). An unpredicted finding was strong binding of the anti-KS mAbs MZ15 and 5D4 to the CSA preparation from bovine trachea. In the original investigations of the specificities of MZ15 and 5D4 interactions with chondroitin sulfates were not observed (41, 42). Those studies were carried out using proteoglycans extracted from bovine nasal cartilage and porcine larynx, respectively. The source of CSA (Sigma C8529) used in our studies is bovine trachea, which is known to contain KS (43). The binding observed with MZ15 and 5D4, as well as R-10G, was suggestive of presence of KS in this polysaccharide. Additional analyses with newly purchased batches of bovine tracheal CSA and shark cartilage CSC from Sigma (supplemental Table S1) show that different batches of these polysaccharides vary in their content of KS immunoreactivity.

**Fig. 2.**
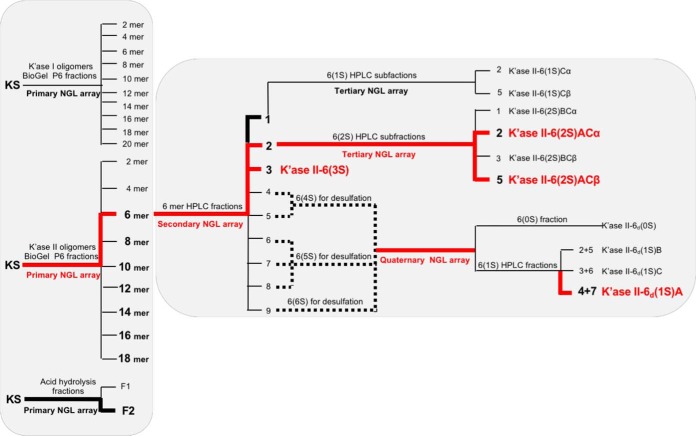
**The Beam Search approach in the assignment of the R-10G antigen.** The primary NGL array encompassed the glycan fractions after partial digestion of KS with keratanase I, keratanase II or mild acid hydrolysis. Fractions bound by mAb R-10G are in bold font. The route that led to assignment of the R-10G antigen is colored red as are the antigen-active glycans assigned.

In sum, our analyses to address the possible KS binding by the anti-stem cell mAbs TRA-1–60, and TRA-1–81 (22) show that neither of these antibodies nor FC10.2 bind to KS or to the other polysaccharides in the array. Among the four anti-stem cell antibodies, only R-10G binds to bovine corneal KS. We selected this polysaccharide to identify and structurally characterize glycan sequence(s) recognized by mAb R-10G by the Beam Search approach (Fig. 2).

##### Preparation of a Primary Beam Search Array of Glycan Fractions of the KS Glycome

The bovine corneal KS was partially depolymerized using keratanases I and II and by mild acid hydrolysis (Experimental Procedures; supplemental Figs. S1 and S2). Ten glycan fractions, 2 to 20 mers of the keratanase I digest, designated K'ase I hereinafter, and nine fractions, 2 to 18 mers of the keratanase II digest, designated K'ase II, were pooled as indicated (supplemental Figs. S2*A* and S2*B*, respectively). Keratanase I is known to cleave the glycosidic bond Galβ-4GlcNAc(6S) to produce glycans with GlcNAc(6S) at their nonreducing ends and Gal at their reducing ends, whereas keratanase II cleaves the glyosidic bond GlcNAc(6S)β-3Gal(±6S) to produce glycans with Gal(±6S) at their nonreducing ends and GlcNAc(6S) at their reducing ends (33, 44). A series of even-numbered glycans were thus produced (Fig. 2).

NMR analyses (supplemental Fig. S3) performed with two tetrasaccharide analogs from K'ase I and K'ase II series (supplemental Table S6) as examples corroborated that the K'ase I products contained glycans with Gal at the reducing ends, and that the K'ase II products contained glycans with GlcNAc(6S) at the reducing ends.

Mild acid hydrolysis of the KS gave rise to a more diverse glycan population (with even and odd monosaccharide numbers) than keratanase treatments. For example, F1 and F2 contained 2–4 mers and 4–7 mers, respectively (supplemental Table S6).

NGLs were generated from the three series of KS glycome fractions for incorporating into a primary Beam Search NGL array. These included the ten K'ase I 2–20 mers, the nine K'ase II 2–18 mers and the two fractions after mild acid hydrolysis (Fig. 2). Also included were NGLs of two synthetic tetrasaccharides, TCI-4(0S) and TCI-4(4S) with no sulfate and four sulfates, respectively (supplemental Table S2).

##### The Minimum Glycan Chain Length of the R-10G Antigen Determined with the Primary Beam Search NGL Microarray

**Fig. 3.**
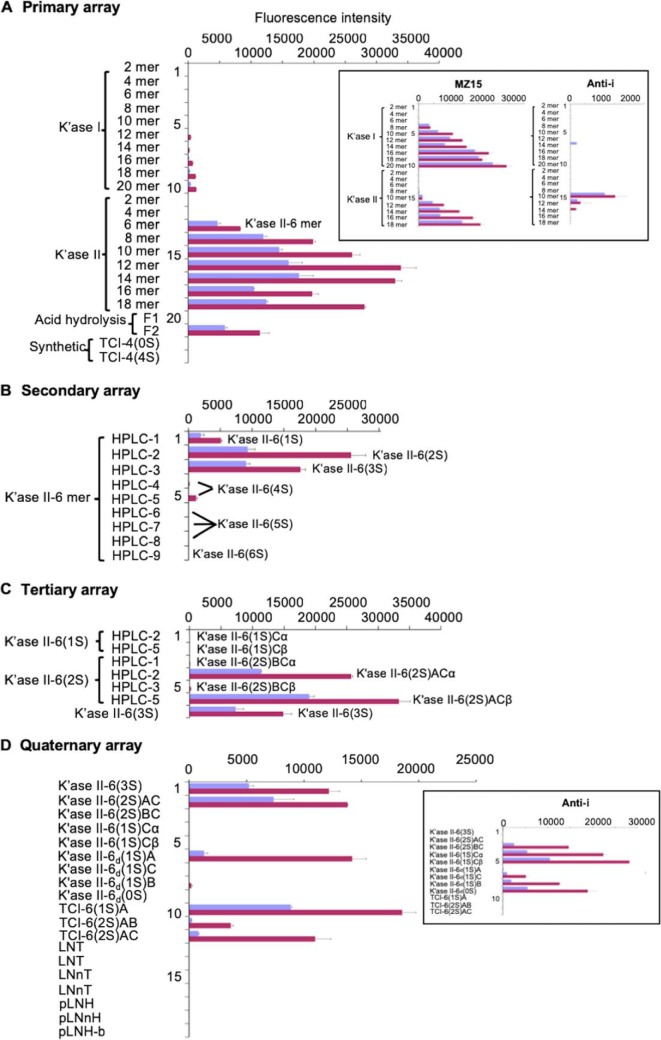
**Microarray analyses of mAbs R-10G, MZ15 and anti-i P1A ELL using the Beam Search NGL arrays.** Results of R-10G binding are presented as histogram charts for primary array, *A*, secondary array, *B*, tertiary array, *C*, and quaternary array, *D*. These are means of fluorescence intensities of duplicate spots, printed at 2 and 5 fmol per spot (blue and red respectively), with error bars representing half of the difference between the values of the duplicate spots. Results of MZ15 and anti-i P1A ELL antibodies are shown as insets in panels *A* and *D*, respectively.

R-10G binding was detected to the NGLs derived from K'ase II-6 mer and longer glycans rather than to those from K'ase I series (Fig. 3*A*). This differed from the pattern of binding by mAb MZ15 (Fig. 3*A*, inset), which bound not only to K'ase II series, 10 mers and longer, but also to the K'ase I series 8 mers and longer. The NGLs of 4–7 mers (F2 after acid hydrolysis) also gave R-10G binding, but there was no binding detected to NGLs of the 4 mer and shorter glycans, F1 (2–4 mers) or the synthetic TCI-4(0S) and TCI-4(4S) (Fig. 3*A*).

Thus the minimum antigen-positive glycan chain length among the K'ase II-derived NGLs was 6 mer. The K'ase II-6 mer fraction, being less heterogeneous than the F2 after acid hydrolysis was selected for generation of the secondary Beam Search array.

##### K'ase II-6 mers with R-10G Antigen Activities Identified and a Tri-sulfated Antigen-positive 6 mer Assigned with the Secondary Beam Search NGL Microarray

**Fig. 4.**
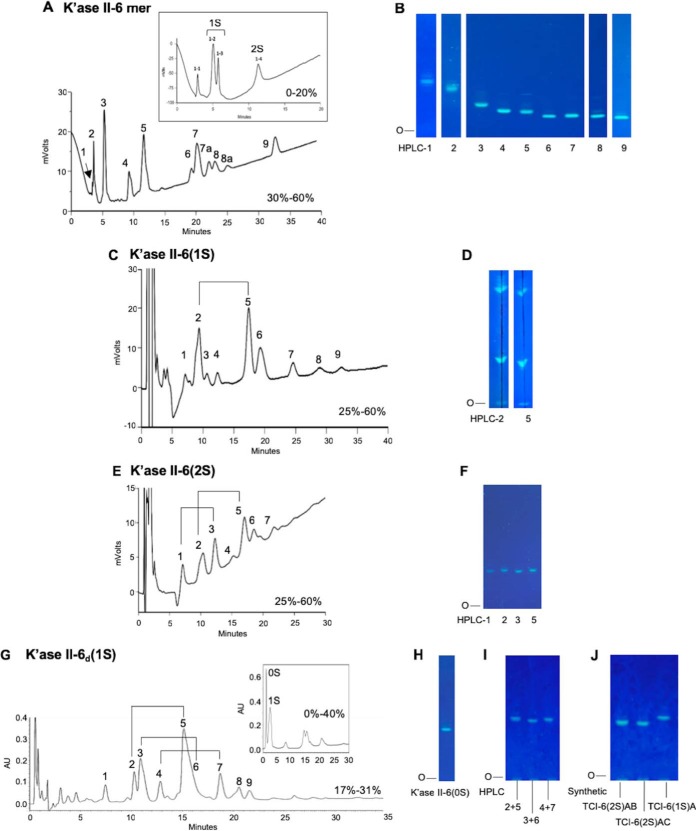
**HPLC of KS 6 mer fractions and HPTLC of the derived NGLs and those of the synthetic 6 mers.** *A*, SAX-HPLC of K'ase II-6 mer fraction; the inset is the further fractionation of the subfraction 1. Peaks 1–2 and 1–3 (mono-sulfated) were pooled and designated 1S. *B*, HPTLC of the NGLs of the 9 fractions indicated in *A. C*, PGC-HPLC of K'ase II-6(1S) fraction. *D*, HPTLC of the NGL reaction mixtures of the 2 fractions indicated in *C. E*, PGC-HPLC of K'ase II-6(2S) fraction; *F*, HPTLC of the NGLs of the 4 fractions indicated in *E. G*, PGC-HPLC of K'ase II-6_d_(1S) fraction; the inset is SAX-HPLC of products of partial desulfation of K'ase II-6(4S+5S+6S) fractions. *H*, HPTLC of the NGL of K'ase II-6(0S). *I*, HPTLC of the NGLs of the three pooled fractions indicated in *G. J*, HPTLC of the NGLs of the three synthetic 6 mers. The percentages in the panels indicate the solvent gradients used for HPLC. NGLs were revealed under UV light with long wavelength after staining with primulin. HPTLC chromatography was in solvent CHCl_3_:MeOH:H_2_O 60/35/8 (v/v/v), and the spaces between figures indicate that they were on separate chromatograms.

The K'ase II-6 mer fraction was subjected to strong anion-exchange (SAX) HPLC to obtain subfractions with different numbers of sulfates. Nine subfractions were harvested (Fig. 4*A*). ESI-MS analyses of these (supplemental Table S7) revealed that HPLC fractions 1, 2, and 3 were mono, di and tri-sulfated 6 mers, respectively; HPLC-4 and 5 were tetra-sulfated 6 mers; HPLC-6, 7 and 8 contained penta-sulfated 6 mers; HPLC-9 contained hexa-sulfated 6 mers; HPLC-7a and -8a were tri- and tera-sulfated 4 mers and penta-sulfated 5 mers, respectively. The nine K'ase II-6 mer subfractions were converted to NGLs (Fig. 4*B*) and included in the secondary array, depicted in Fig. 2.

**Fig. 5.**
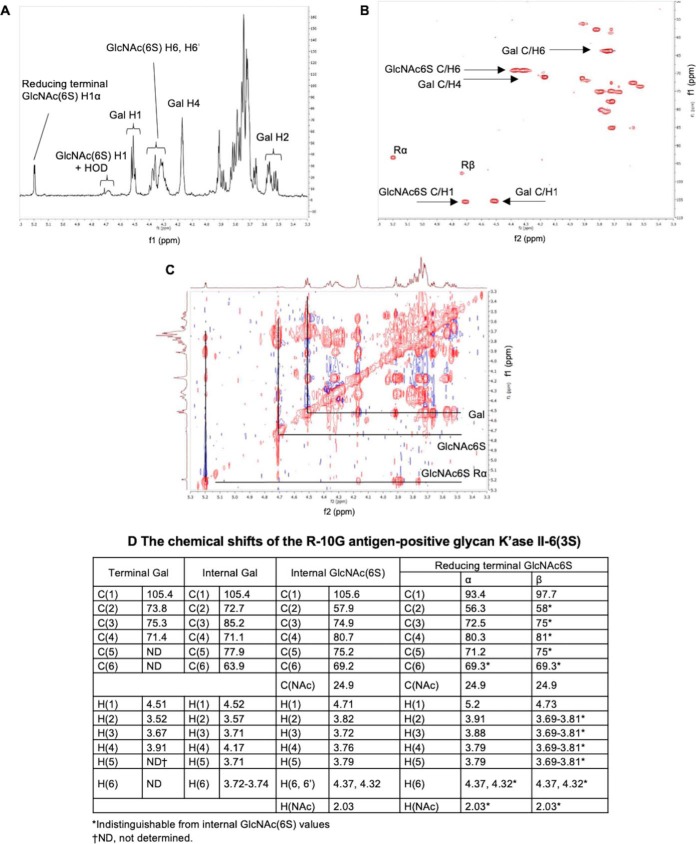
**NMR data on the R-10G antigen-positive K'ase II-6(3S).** *A*, ^1^H spectrum at 700 MHz; *B*, HSQC spectrum; *C*, TOCSY spectrum; *D*, chemical shifts.

The tri-sulfated K'ase II-6 mer fraction (HPLC-3) was strongly bound by R-10G (Fig. 3*B*). The ^1^H, ^1^H-^1^H TOCSY and ^1^H-^13^C HSQC 2D spectra of the fraction are shown in Fig. 5*A*, 5*B*, and 5*C*, respectively. The values in Fig. 5*D* were determined by comparison of HSQC cross-peak chemical shift (Fig. 5*B*) with those in the literature (34, 45, 46), with confirmation by correlations in the TOCSY spectrum (Fig. 5*C*). Chemical shift values for reducing end residues of KS oligosaccharides have not been published previously, so the reducing end α-GlcNAc6S anomer residue was assigned by comparison with 4-linked α-GlcNAc6S in modified heparins (47, 48) with confirmation by correlations in the TOCSY spectrum (Fig. 5*C*). The spectrum of reducing end β-GlcNAc6S anomer was, apart from the anomeric center, indistinguishable from the internal β-GlcNAc6S. No signals attributable to nonsulfated GlcNAc (49) were present. The R-10G antigen-positive glycan was thus established as the tri-sulfated hexasaccharide shown in Table III, and designated K'ase II-6(3S).

**Table III:**
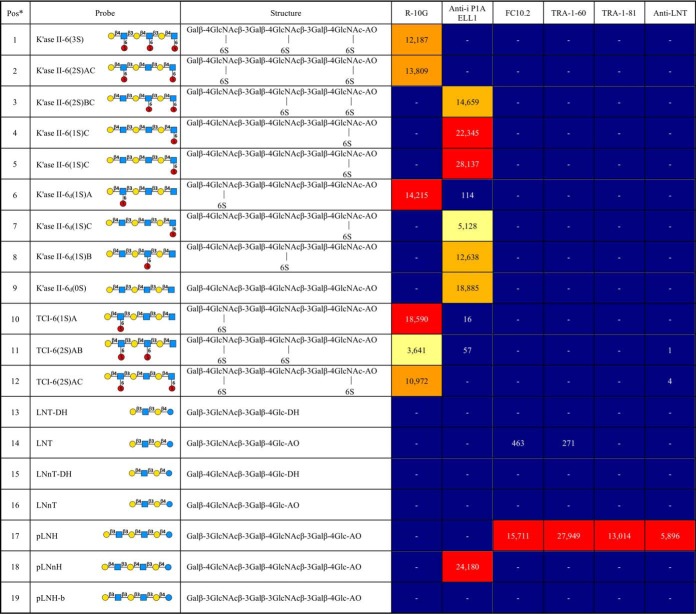
Relative intensities of binding of mAbs R-10G, anti-i P1A ELL, FC10.2, TRA-1–60, TRA-1–81 and anti-LNT to the NGL probes in the quaternary NGL array 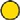
Gal, galactose; 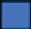
GlcNAc, *N*-acetylglucosamine; 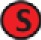
sulfate(6S), sulfate at position 6 of GlcNAc. Binding scores are means of the fluorescence intensities at 5 fmol/probe spot; - indicates the score is less than 1. Relative binding intensity: 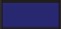
< 10%, 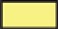
10–30%, 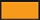
30–70%, 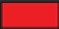
70–100%, where 100% is the maximum score for respective antibodies.

^a^ Pos, position in the microarray.

The di-sulfated K'ase II-6 mer fraction (HPLC-2), K'ase II-6(2S), was also strongly bound (Fig. 3*B*) but it was a mixture (Fig. 4*E*). There was binding albeit weak to the mono-sulfated K'ase II-6 mer fraction (HPLC-1), K'ase II-6(1S) (Fig. 3*B*), which was also a mixture (Fig. 4*C*). There was little or no binding to the more highly sulfated K'ase II-6 mers with 4 sulfates (HPLC-4 and 5), 5 sulfates (HPLC-6, 7, and 8) and 6 sulfates (HPLC-9), which are designated K'ase II-6(4S), K'ase II-6(5S) and K'ase II-6(6S), respectively (Fig. 3*B*).

Collectively, these data suggest that R-10G antigen is expressed not only on tri-sulfated 6 mer, K'ase II-6(3S), but also on di and mono-sulfated 6 mers, K'ase II-6(2S) and K'ase II-6(1S); but not on 6 mers with more than three sulfates.

##### The Positions of Sulfate on R-10G Antigen-positive and -negative Di-sulfated K'ase II-6 mers Assigned with the Tertiary Beam Search Microarray

The antigen-positive di- and mono-sulfated fractions, K'ase II-6(2S) and K'ase II-6(1S), were subjected to further fractionation by HPLC using a PGC column. Seven subfractions were obtained from the K'ase II-6(2S), of which four (HPLC-1, 2, 3, and 5) were in sufficient amounts for NGL preparation and antigenic analysis (Fig. 4*E* and 4*F*). Nine subfractions were obtained from K'ase II-6(1S), of which two (HPLC-2 and 5) were enough for NGL preparation and antigenic analysis (Fig. 4*C* and 4*D*). These six NGLs were arrayed as the tertiary array and together with the K'ase II-6(3S) constituted supplements to the secondary array (Fig. 3*C*).

**Fig. 6.**
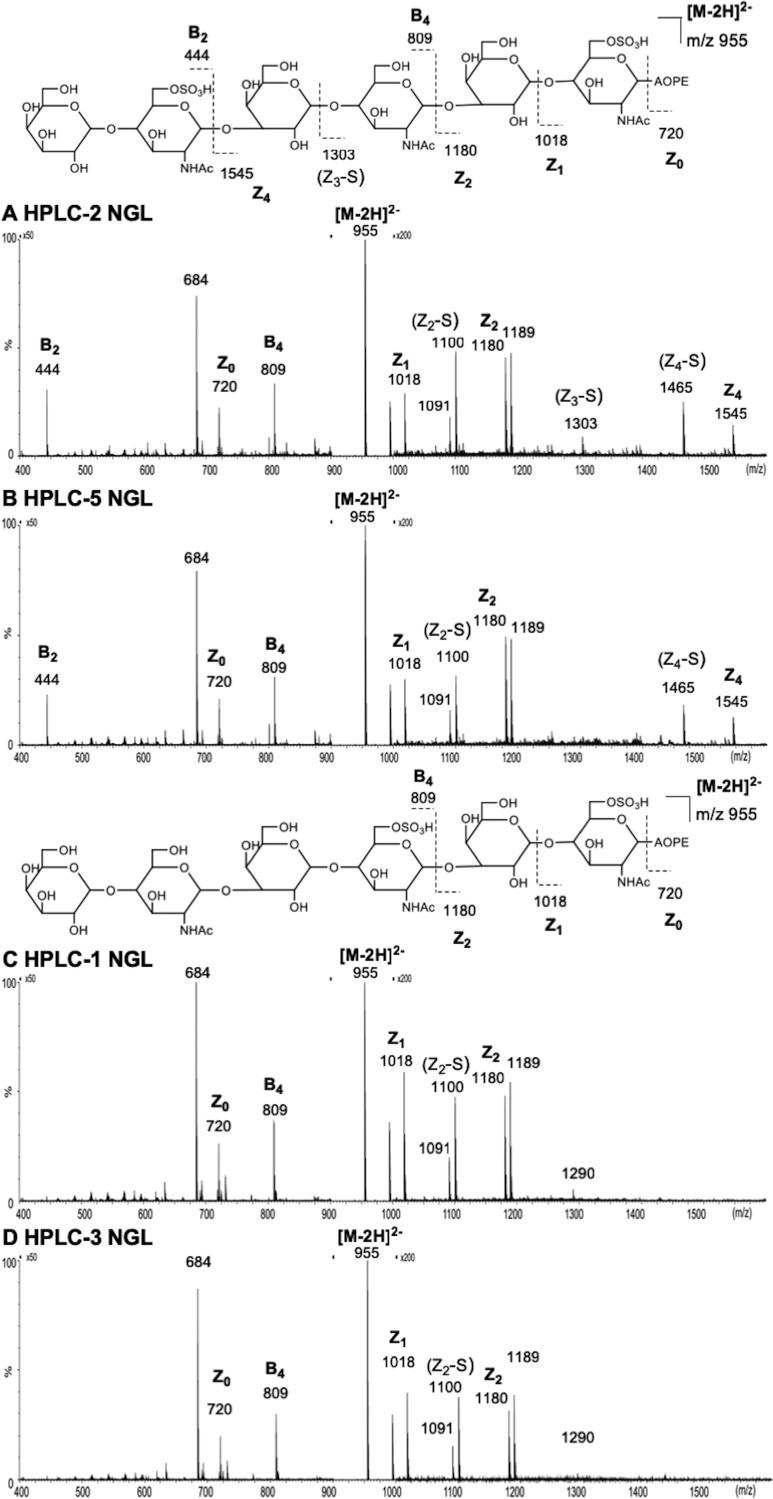
**ESI-CID-MS/MS product-ion spectra of the NGLs of glycans derived from fraction K'ase II-6(2S).** *A, B, C,* and *D* are NGLs of HPLC-2, HPLC-5, HPLC-1 and HPLC-3, respectively.

**Fig. 7.**
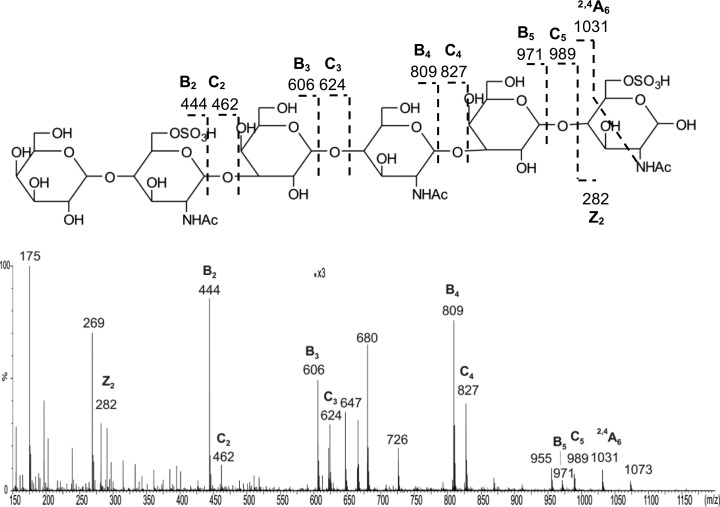
**ESI-CID-MS/MS product-ion spectrum of the underivatized R-10G antigen-positive glycan from HPLC-5.**

Subfractions HPLC-2 and 5 of the K'ase II-6(2S) fraction elicited R-10G binding, but HPLC-1 and 3 did not (Fig. 3*C*). ESI-CID-MS/MS analysis of the two R-10G antigen-positive NGLs from the di-sulfated HPLC-2 and 5 gave almost identical product-ion spectra using [M-2H]^2−^ as the precursors (Fig. 6*A* and 6*B*), indicating a pair of anomeric isomers. Although, because of the presence of two sulfate groups the fragmentation is rather complex, making the assignment very difficult, it is still possible to identify the location of the two sulfates using some key fragment ions. One sulfate at the reducing terminal GlcNAc is apparent with the sulfated Z_1_ ion (*m/z* 1018) and further corroborated by the Z_2_ (*m/z* 1180) (nomenclature of fragmentation see (50)). The latter ion also indicated the absence of the sulfate on the Gal residue. The other sulfate can also be unambiguously assigned at the nonreducing terminal GlcNAc with the unique sulfated B_2_ ion at *m/z* 444. The product-ion spectrum of one of the anomers, HPLC-5, as free sugar (Fig. 7) before NGL conjugation can be used to make the assignment of the active sequence as some additional fragment ions were present, although the spectrum is more complex because of the sulfate-related fragmentation. A sulfate at the nonreducing terminal GlcNAc again can be deduced from the B_2_ (*m/z* 444) and C_2_ (*m/z* 462) ions and the other sulfate at the reducing terminal GlcNAc by B5 (*m/z* 971) and C5 (*m/z* 989) ions. The lack of a sulfate at either internal or reducing terminal Gal residues was evidenced by the B_3_ and C_3_ (*m/z* 606 and 624), and B_5_ and C_5_ (*m/z* 971 and 989) ion pairs. The ^2,4^A_6_ ion at *m/z* 1031 suggested the sulfate at the 6-O-position. HPLC-2 and 5 are thus assigned as the α and β anomeric forms, respectively, of the same glycan sequence. The sequence of the two antigen-positive glycans was deduced and theses were designated K'ase II-6(2S)AC (Fig. 2). Their sequences are shown in Table III. We designate the GlcNAc residues at the nonreducing, middle and reducing ends as A, B and C, respectively.

The spectra of the two antigen-negative NGLs from HPLC-1 and -3 are also very similar (Fig. 6*C* and 6*D*) and HPLC-1 and -3 were considered as anomeric isomers as discussed above. These two spectra are different from those of the antigen-positive fractions HPLC-2/5. The presence of Z_1_ (*m/z* 1018) and Z_2_ (*m/z* 1180) ions indicated the location of the sulfate at the reducing terminal GlcNAc. The lack of a sulfate at the nonreducing terminal GlcNAc is evidenced by the absence of the B_2_ ion at *m/z* 444. The second sulfate is therefore deduced to be at the internal GlcNAc. HPLC-1 and 3 are then assigned as α and β anomers, respectively, of K'ase II-6(2S)BC (Fig. 2) The sequence is shown in Table III.

For the two mono-sulfated antigen-negative subtractions, HPLC-2 and 5 of the K'ase II-6(1S) (Figs. 3*C* and 4*C*), the positions of sulfate could not be assigned as NGLs. As the antigenic analyses with anti-i P1A ELL using in the quaternary array (below) indicated that the sulfate is not at position A of the GlcNAc residues, and taking that into account the cleavage specificity of K'ase II for the GlcNAc(6S)-Gal sequence, these two glycans were assigned the α and β anomeric forms of the same glycan K'ase II-6(1S)C (Fig. 2).

The main conclusion that could be drawn from the tertiary array analysis was that the K'ase II-6(2S)AC with sulfate at the nonreducing and reducing end GlcNAc residues is R-10G antigen-positive whereas the analog K'ase II-6(2S)BC with sulfate at the internal and reducing end GlcNAc residues is antigen-negative. This highlighted the need to have a mono-sulfated 6 mer K'ase II-6(1S)A with sulfate at position A not only to determine whether this is sufficient for strong R-10G antigenicity but to rule out any contribution of sulfate at the internal position (position C) to the strong antigenicity of K'ase II-6(3S) and K'ase II-6(2S)AC.

##### A Mono-sulfated 6 mer K'ase II-6(1S)A Assigned as the R-10G Antigen with a Quaternary Beam Search Microarray and Corroborated with Synthetic Glycans

The mono-sulfated K'ase II-6(1S) fraction was antigen-positive (Fig. 2 and 3*B*). However, this fraction was heterogeneous and obtained in low yield. Only two of the subfractions (they are considered α and β anomers) were in sufficient amounts for antigenic analysis; both were R-10G antigen-negative (Figs. 2 and 3*C*). To have sufficient amounts of mono-sulfated 6 mers, we pooled all of the over-sulfated (tetra-, penta- and hexa-sulfated) 6 mer glycan fractions and carried out partial desulfation of these, as depicted in Fig. 2.

**Fig. 8.**
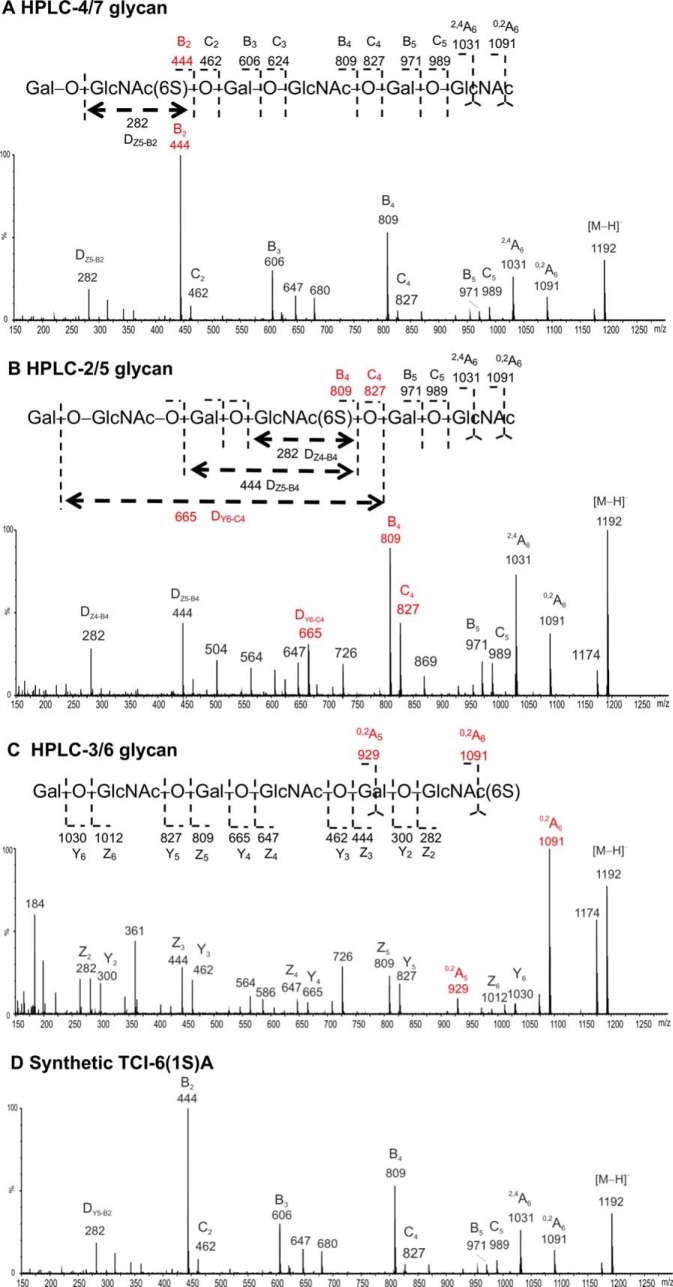
**ESI-CID-MS/MS product-ion spectra of the glycans K'ase II-6_d_(1S)A, B and C (Panels *A*, *B,* and *C*, respectively), isolated from the de-sulfated hexamers substituted with 4 to 5 sulfates, compared with the spectrum of the synthetic TCI-6(1S)A (Panel *D*).**

The partially de-sulfated fractions were separated into a nonsulfated [designated as K'ase II-6_d_(0S)] and a mono-sulfated fraction with a SAX column (Fig. 4*G* inset); the subscript d indicates a de-sulfated product. The mono-sulfated fraction was then subfractionated with a PGC column into nine subfractions (Fig. 4*G*). The anomeric pairs HPLC-2/5, 3/6 and 4/7 (Fig. 4*G*) of the three mono-sulfated sequence isomers were assigned by ESI-CID-MS/MS and by HPLC. The sequence of the R-10G antigen positive mono-sulfated KS 6 mer HPLC fraction (HPLC-4/7), before NGL conjugation, was assigned by ESI-CID-MS/MS using [M-H]^−^ as the precursor (Fig. 8*A*). The location of the sulfate at the nonreducing terminal side GlcNAc is indicated by the glyosidic fragment B_2_ (*m/z* 444), C_2_ (*m/z* 462), and the double-cleavage D_Y5-B2_ ion (*m/z* 282). The exclusive nonreducing terminal fragments B_3_/C_3_, B_4_/C_4_ and B_5_/C_5_ and the absence of B_1_/C_1_ also suggested the sulfate at the sub-terminal (nonreducing end) GlcNAc. The product-ion spectra of the antigen-negative fractions HPLC-2/5 and HPLC-3/6 are very different from those of HPLC-4/7 (Fig. 8*B* and 8*C*). A product-ion spectrum of a synthetic mono-sulfated 6 mer TCI-6(1S)A with the sulfate at the nonreducing sub-terminal position was also acquired and was identical to that of HPLC-4/7.

The sequence of HPLC-4/7 was thus assigned to the desired analog with sulfate at positions A, designated as K'ase II-6_d_(1S)A (Sequence shown in Table III). The sequence of HPLC-2/5 was assigned as below and is designated as K'ase II-6_d_(1S)B (Table III). The sequence of HPLC-3/6 was assigned as below and designated as K'ase II-6_d_(1S)C (Table III). The nonsulfated 6 mer K'ase II-6_d_(0S) and the purified three mono-sulfated 6 mers, K'ase II-6_d_(1S)A, K'ase II-6_d_(1S)B and K'ase II-6_d_(1S)C were converted to NGLs (Figs. 4*H* and 4*I*, respectively). These NGLs were included in the quaternary array (Fig. 2), together with NGLs of K'ase II-6(3S), K'ase II-6(2S)AC, K'ase II-6(2S)BC and K'ase II-6(1S)C. Also included as reference compounds were NGLs of LNT, LNnT, pLNH, pLNnH and pLNH-b (supplemental Table S2 and Fig. 3*D*). To verify the binding data with the KS-derived hexasaccharides, three of the sequences considered important in assignment of the R-10G antigen were custom synthesized chemically at TCI. These were the mono-sulfated and two di-sulfated 6 mers, designated as TCI-6(1S)A, TCI-6(2S)AB and TCI-6(2S)AC, respectively (supplemental Table S2). These were converted to NGLs (Fig. 4*J*) for inclusion in the quaternary array (Fig. 3*D*).

Of the three mono-sulfated 6 mers derived from the de-sulfated fraction, K'ase II-6_d_(1S)A elicited strong R-10G binding as did TCI-6(1S)A and TCI-6(2S)AC (Figs. 3*D* and Table III). The binding signal was similar to those of the tri- and di-sulfated, K'ase II-6(3S) and K'ase II-6(2S)AC, respectively. This established that the 6 mer with a sulfate at the nonreducing terminal position (position A) is sufficient for and constitutes the R-10G antigen.

##### A Revisit of mAbs FC10.2, TRA-1–60, and TRA-1–81 and the Anti-i Using the Quaternary NGL Microarray to Compare Binding Profiles with that of mAb R-10G

Advantage was taken of the Beam Search arrays in the context of reference glycans to analyze the three anti-stem cell antibodies FC10.2, TRA-1–60, TRA-1–81 (Table III) also the anti-i (Table III, and Fig. 3), compared with R-10G (Table III). There was no binding of R-10G to the six nonsulfated NGL reference compounds (Fig. 3*D*). The distinct binding specificities of FC10.2, TRA-1–60 and TRA-1–81 toward the T1-T2-T2 sequence were corroborated. A new observation made possible with the relatively recently generated NGL of the pLNH-b (T1-T2-T1 sequence), isomer of pLNH (51), is the lack of binding of FC10.2, TRA-1–60 and TRA-1–81 to this hexasaccharide sequence with an inner T1 chain (Table III).

An interesting reciprocal pattern of binding was observed for the anti-i with the present series of under-sulfated KS glycans. It is known that in fully sulfated KS (*i.e.* on all Gal and GlcNAc residues), the i-antigen is masked (36). Here we found a lack of binding of anti-i P1A ELL to all of the R-10G antigen-positive 6 mers with sulfate on GlcNAc at position A whereas there was strong binding to those with sulfate at positions B and C (Fig. 3*D* inset and Table III). Thus, anti-i P1A ELL can accommodate sulfate substituents on GlcNAc at positions B and C (Table III). The two minor subfractions (HPLC-2 and -5) of K'ase II-6(1S), which were R-10G antigen-negative but could not be sequenced by MS, were strongly bound by the anti-i (Table III). We used this immunochemical information as evidence that sulfate is not at position A, and assigned the sequence as K'ase II-6(1S)C.

##### Binding of K'ase II-6 mers with 4, 5, or 6 Sulfate Groups by FGF2 and BMP-2 Detected in Exploratory Microarray Analyses

Prompted by the observations in ref (52). on the binding of human fibroblast growth factor FGF2 and the morphogen Sonic hedgehog (Shh) to KS polysaccharide, we performed exploratory microarray analyses of FGF1 and FGF2, Shh, int/wingless (Wnt)-3a, bone morphogenetic proteins, BMP-2 and BMP-4, using the secondary NGL array and the results are shown in Table IV. We detected binding of FGF2 and BMP-2 to KS glycan populations. Among the K'ase II-6 mers there was detectable binding to those with 4, 5, or 6 sulfate groups designated HPLC-4, 5, 6, 7, 8, and 9, but not to those with less than 4 sulfate groups, HPLC-1, 2, and 3, which were bound by R-10G (supplemental Table S7).

**Table IV:**
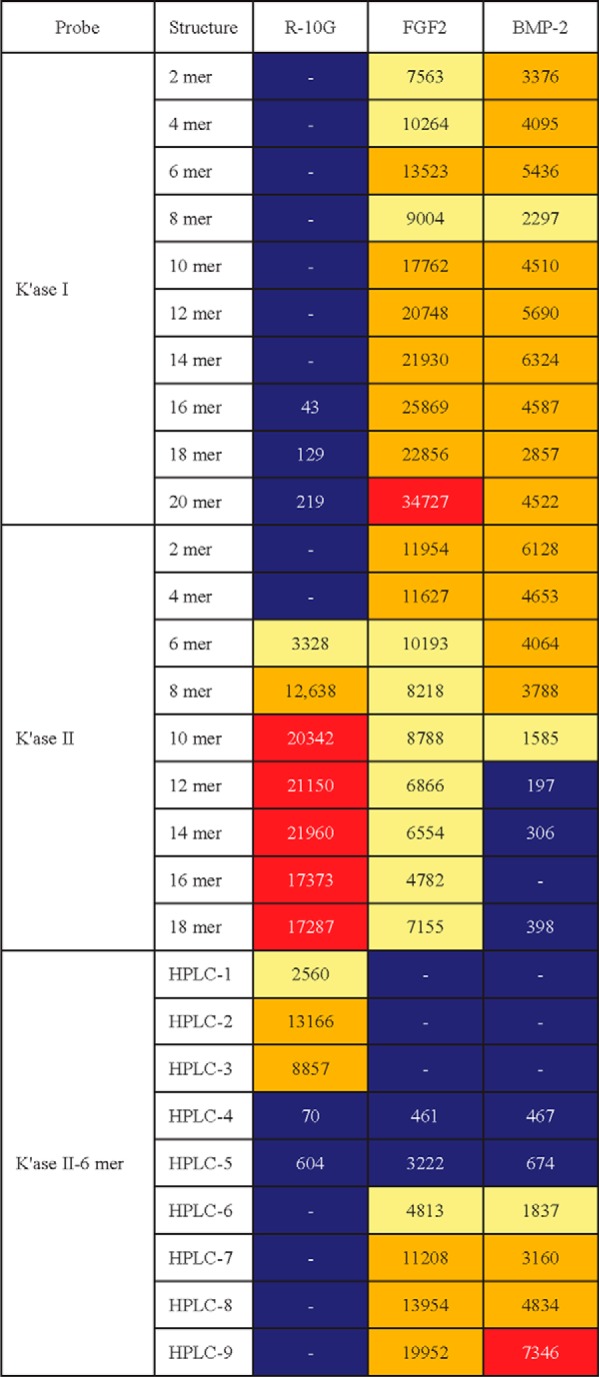
Relative intensities of binding of mAb R-10G, growth factor FGF2 and morphogen BMP-2 in secondary NGL microarray Binding scores are means of the fluorescence intensities at 5 fmol/probe spot; - indicates the score is less than 1. Relative binding intensity: 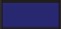
< 10%, 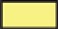
10–30%, 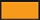
30–70%, 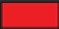
70–100%, where 100% is the maximum score for respective antibodies. Growth factor FGF1 and morphogens Wnt-3a, Shh and BMP-4 were analyzed in the microarray but no binding was detected.

## DISCUSSION

Knowledge of the glycan sequences and substituents recognized by anti-stem cell antibodies has an important bearing on their use as reagents to gain insights into the glycans displayed, and the changes that occur on differentiation. As more sequence-defined glycan probes become available the antibody specificities can be reassessed. Our studies here contribute to the refinement of the assignments of the binding specificities of five mAbs that recognize human stem cells. They provide new insights into the antigen recognized by the more recently described mAb R-10G.

### 

#### 

##### The mAbs FC10.2, TRA-1–60, and TRA-1–81 Have A Determinant In Common but FC10.2 and TRA-1–60 Differ In Their Ability To Accommodate A Fucose Substituent

We have now closely compared for the first time the three well-established anti-stem cell antibodies, FC10.2, TRA-1–60, and TRA-1–81, and shown that they are very similar in their strong binding to the nonsubstituted T1-T2 sequences as part of linear or branched glycan backbones that are longer than tetrasaccharide. No binding was detected to any of the sulfated glycans arrayed. The tolerance of a fucose 3-linked to the inner GlcNAc by FC10.2, TRA-1–60 but not by TRA-1–81 would be predicted to give rise to differences in immunostaining of certain tissues that contain this fucosylated sequence.

##### The R-10G Antigen Is A Unique Mono-sulfated Glycan Antigen

Under the analysis conditions used, no overlap was observed in the binding specificities of mAbs FC10.2, TRA-1–60, TRA-1–81 and anti-i P1A ELL with that of mAb R-10G. In the initial investigations of the R-10G determinant expressed on podocalyxin, the antigen was suggested to be on *O*-glycans without sialic acid and fucose (19). Evidence of sulfate involvement in the R-10G antigen was obtained: the main product after keratanase II digestion was assigned as the disaccharide Gal-GlcNAc(S) (19); the presence of additional glycans such as Gal-GlcNAc(S)-Gal-GlcNAc(S) was suggested as minor components. In a follow up study (21) the chemically synthesized di-sulfated tetrasaccharide below, in biotinylated from was shown to be bound by mAb R-10G.

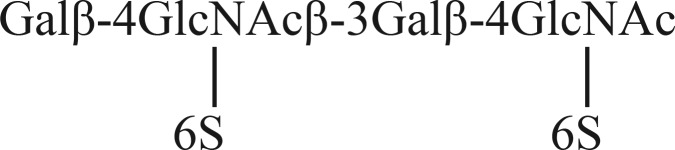


In the present study, our finding of R-10G binding to the tri-sulfated hexasaccharide below isolated from KS via the Beam Search approach is in complete accord with the earlier studies (21).

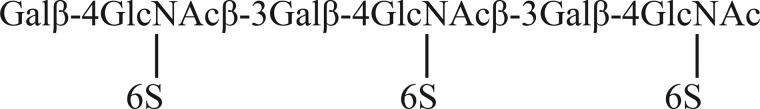


A second antigen-positive glycan, di-sulfated at positions A and C (as below) was also isolated

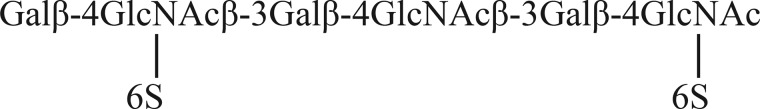


R-10G activity was additionally detected in the heterogeneous unfractionated mono-sulfated fraction, K'ase II-6(1S), but the glycan could not be isolated in sufficient amount for antigenic and structural analysis. Considering the cleavage specificity of keratanase II, it is likely that the antigen-active component was a trace contaminant from the adjoining K'ase II-6(2S) fraction. This prompted the partial desulfation strategy to create a sufficient amount of the mono-sulfated antigen below for sequence determination.

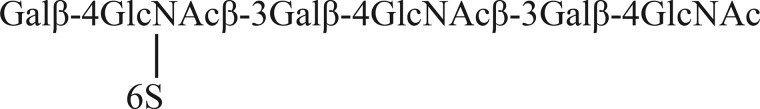


We thereby demonstrated that sulfation at either the B or C position is not required for antigenicity. We propose that the A analog is a low abundance capping sequence on the model saccharide we have used (corneal KS) (53), and that it is among A, AB, AC and ABC hexasaccharide forms on the podocalyxin on stem cells. The relative amounts in ES and iPS cells may well differ. These need investigation in future studies.

We conclude, based on the earlier (19) and current studies, that the chain length of the R-10G antigen is between a 4 mer and 6 mer. When presented as a NGL the minimum length bound is a 6 mer, although in a biotinylated form [associated with a larger spacer length (54)] binding is detectable with a 4 mer. Moreover, the lack of R-10G binding to the series of GlcNAc-terminating glycans (irrespective of chain length) generated by keratanase I treatment of the KS lead us to conclude that the R-10G epitope is necessarily at the nonreducing end rather than a part of an internal sequence. The effect of sulfation of the Gal nonreducing on R-10G binding requires investigation.

##### Influence of Sulfation on i Antigen Expression and the Reciprocal Relationship with R-10G

**Scheme 1:**
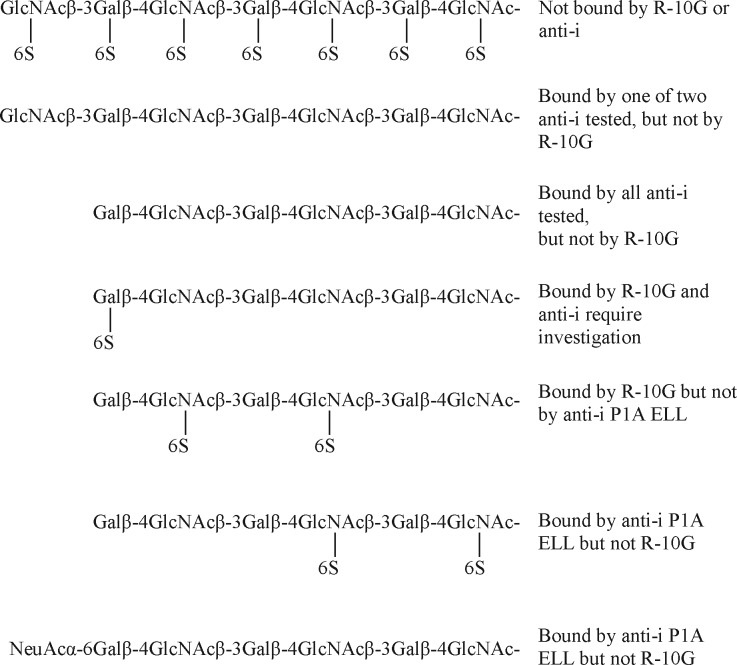


In the light of the reported expression of i antigen on human mesenchymal stem cells (55), it was opportune to investigate the effect of sulfation on its expression. Earlier studies from our group (36) showed that i antigen expression on the unbranched T2-T2 sequence is masked on KS chains that are fully 6-sulfated on Gal and GlcNAc residues. As depicted overleaf (Scheme 1) we also showed that the antigen is revealed after total desulfation and that different anti-i differ in their ability to bind to the de-sulfated KS glycans that have GlcNAc at their nonreducing ends. We have now observed that on the KS-derived hexasaccharide with a single 6-linked sulfate on the GlcNAc at position A recognized by mAb R-10G expression of i antigen as seen by anti-i P1A ELL is completely masked, but not when 6-linked sulfate is on GlcNAc at positions B or C or on both (Scheme 1). Thus, there is a reciprocal expression of anti-i P1A ELL and R-10G.

Whether 6-linked sulfate on the Gal at the nonreducing end influences the i antigen expression will be interesting to determine in the light of the strong binding of anti-i P1A ELL in the presence of 6-linked sialic acid at this position [this has been observed recently with other anti-i (Zhen Li and Chao Gao, unpublished)]. We have not investigated here the i antigen expression on glycans with 6-sulfation at this position. But we observe weak binding signals with NGLs of the K'ase II 10–14 mer fractions where the nonreducing terminal Gals are variably sulfated (34), whereas there are strong signals detected with several of the NGLs in the quaternary array where there is no sulfation at this position. Collectively our data suggest that 6-linked sulfate at the nonreducing terminal Gal masks the i determinant recognized by the anti-i P1A ELL. There may be analogies here with proteins of the innate immune system known as siglecs that have a requirement for terminal sialic acids and fail to recognize the sulfated analogs. This is in contrast with the selectins that bind equally well to the sulfated and sialylated analogs (56). In the light of the status of i antigen as a marker of on human mesenchymal stem cells (55), the effect of sulfation of the terminal Gal on the expression of i requires investigation with a series of ant-i antibodies.

##### Relationship of R-10G Antigen and the Glycan Ligand of L-selectin

**Scheme 2:**
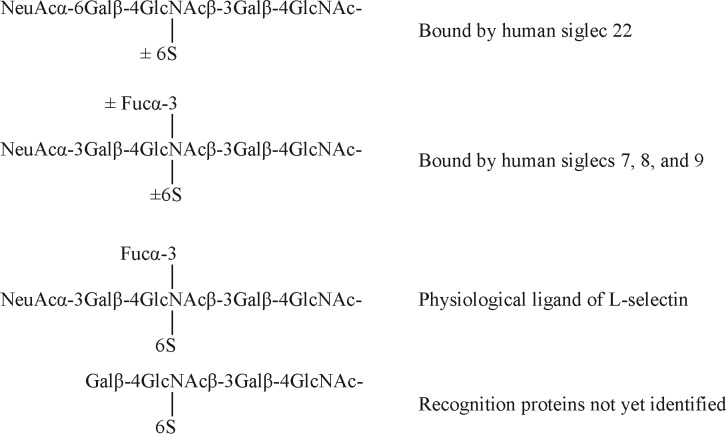


The mono-sulfated R-10G sequence, with the sulfate at position A, is a constituent of glycan sequences shown by microarray analysis to be bound by human siglec 22 (57) and human siglecs 7, 8, and 9 (56), as depicted below (Scheme 2), but for all of the four proteins the presence of the sialic acid residues is essential. The sequence A in sialylated and fucosylated form is the physiological ligand on high endothelial venules for the lymphocyte homing receptor, L-selectin; here the presence of 6-linked sulfate (at A) is essential (58) (Scheme 2). One of the carrier glycoproteins (counter-receptors) of the L-selectin ligand is a podocalyxin like glycoprotein on high endothelial venules (59). A further analogy with the L-selectin ligand is the indication [based on resistance to PNGase treatment (19)] that the R-10G determinant is borne on *O*-glycans. We propose that the unique R-10G determinant be considered a member of the L-selectin ligand family for which it will be exciting to search for a counter-receptor(s) in the ES and iPS cell niche.

##### Potential Roles of the Glycan Antigens on Stem Cells In Signaling

The biological aspects of the several human stem cell-associated glycan sequences including the R-10G antigen indeed warrant detailed exploration. As discussed in the INTRODUCTION many of them are expressed on the highly glycosylated molecule podocalyxin and related glycoproteins. Podocalyxin and related glycoproteins such as CD34 are cell signaling molecules (60, 61). New cell biological avenues would open by discoveries of proteins (counter receptors) that interact with the glycans and thereby cluster the carrier glycoproteins or form parts of complexes that regulate the dormant or regenerative preparties and fate of the stem cells. In addition, detailed studies are required of the regulation of the activities not only of glycosyltransferases but also of sulfotransferases (62) during stem cell differentiation. There may be analogies with changes in sulfation of HS during stem cell differentiation (63, 64). Moreover, bespoke binding as well as kinetical analysis systems require design to gain insights into recognition of sequentially changing glycan displays on stem cells by growth factors and morphogens.

## Data Availability

Mass spectral data have been deposited to JPOST (https://repository.jpostdb.org/) with the dataset identifier (JPOST ID) 'JPST000605'.

## Supplementary Material

supplemental Table S2

Supplemental data

## Footnotes

^1^ The abbreviations used are:

ES: embryonic stem
AOPE: *N*-aminooxyacetyl-1,2-dihexadecyl-*sn*-glycero-3-phosphoethanolamine
BMP: bone morphogenetic proteins
CS: chondroitin sulfates
EC: embryonal carcinoma
FGF: fibroblast growth factor
GAG: glycosaminoglycan
Gal: galactose
GalNAc: *N*-acetylgalactosmine
Glc: glucose
GlcNAc: *N*-acetylglucosamine
iPS: induced pluripotent
KS: keratan sulfate
MIRAGE: minimum information required for a glycomics experiment
NGL: neoglycolipid
RI: refractive index
SAX: strong anion-exchange
Shh: sonic hedgehog
sulfate(6S): sulfate at position 6 of GlcNAc
Wnt: int/wingless.
