# Supplementary material for: Glycan Markers of Human Stem Cells Assigned with Beam Search Arrays
Source: Mol Cell Proteomics. 2019 Jul 15;18(10):1981–2002. doi: 10.1074/mcp.RA119.001309 (PMC6773554; doi:10.1074/mcp.RA119.001309)
Supplement: supplemental Table S2 [file RA119.001309_index.html]

Supplement to Glycan Markers of Human Stem Cells Assigned with Beam Search Arrays | Molecular & Cellular Proteomics

## Supplemental Data

- Supplemental data - Supplemental tables and figures
