# Supplementary material for: Glycan Markers of Human Stem Cells Assigned with Beam Search Arrays
Source: Mol Cell Proteomics. 2019 Jul 15;18(10):1981–2002. doi: 10.1074/mcp.RA119.001309 (PMC6773554; doi:10.1074/mcp.RA119.001309)
Supplement: Supplemental data [file 142607_1_supp_351529_ptg4cy.docx]

**Glycan Markers of Human Stem Cells Assigned with Beam Search Arrays**

Nian Wu^1#^, Lisete M. Silva^1^, Yan Liu^1^, Yibing Zhang^1^, Chao Gao^1,4^ Fuming Zhang^2^, Li Fu^2^, Yanfei Peng^2^, Robert Linhardt^2^, Toshisuke Kawasaki^3^, Barbara Mulloy^1^, Wengang Chai^1*^ and Ten Feizi^1*^

**SUPPLEMENTAL DATA**

1. **SUPPLEMENTAL FIGURES**

**Figure S1. Chromatography on Superdex Peptide column of aliquots of bovine corneal KS depolymerized with keratanase I at 5, 8 and 22 h, panel *A*, keratanase II at 2, 5 and 6.5 h, panel *B*, and mild acid at 2 and 4 h, panel *C***

**Figure S2. Chromatographs of the KS derived oligosaccharides after partial digestion with keratanase I, panel *A*, keratanase II, panel *B*, and mild acid, panel *C***

**Figure S3. Anomeric region of the HSQC NMR spectra of tetrasaccharide fractions obtained from partial digestion of KS with keratanase I, panel *A*, and keratanase II, panel *B***

1. **SUPPLEMENTAL TABLES**

**Table S1. Relative binding intensities of mAbs MZ15 and CS56 with commercial CSA and CSC in microarray analysis**

**Table S2. Supplemental glycan microarray document based on MIRAGE guidelines**

**Table S3. Sequences of glycan probes in CLL microarray**

**Table S4. Results of binding of mAbs R-10G, anti-i P1A ELL, FC10.2, TRA-1-60, TRA-1-81 and anti-LNT to glycan probes in CLL glycan microarray set**

**Table S5. Quality control data for CLL glycan microarray set: results of binding of selected plant lectins and anti-carbohydrate antibodies**

**Table S6. Negative-ion ESI-MS analysis of glycans up to heptasaccharides obtained after size fractionations of bovine corneal KS following keratanase I or keratanase II partial digestions or mild acid treatment**

**Table S7. Negative-ion ESI-MS analysis of glycan subtractions obtained by SAX-HPLC of K’ase II-6 mer fraction and of the derived NGLs**

**1. SUPPLEMENTAL FIGURES**

**Figure S1. Chromatography on Superdex Peptide column of aliquots of bovine corneal KS depolymerizd with keratanase I at 5, 8 and 22 h, panel *A*, keratanase II at 2, 5 and 6.5 h, panel *B*, and mild acid at 2 and 4 h, panel *C*.** Positions of elution of glucose oligomers; GU, glucose units with degrees of polymerization 1-11; V_0_ (void volume).

**Figure S2. Chromatographs of the KS derived oligosaccharides after partial digestion with keratanase I, panel *A*, keratanase II, panel *B*, and mild acid, panel *C*.** Panels *A* and *B* were on Bio-Bio-Gel P6 and ten and nine fractions were collected, respectively; *C* was on Superdex Peptide and three fractions were pooled indicated.

**Figure S3. Anomeric region of the HSQC NMR spectra of tetrasaccharide fractions obtained from partial digestion of KS with keratanase I, panel *A*, and keratanase II, panel *B*.** NR and internal means non-reducing and internal residues. Proton chemical shifts of the cross-peaks indicate that *A* has Gal at its reducing end whereas *B* has GlcNAc at its reducing end.

**2. SUPPLEMENTAL TABLES**

**Table S1. Relative binding intensities of mAbs MZ15 and CS56 with commercial CSA and CSC in microarray analysis**

| Pos. | Polysaccharides | MZ15 | CS56 | Composition |
| --- | --- | --- | --- | --- |
| 1 | HA | 9 | 98 | Bovine vitreous humor, Sigma H7630 |
| 2 | KS | 17,679 | 38 | Bovine cornea ([Weyers et al., 2013](#_ENREF_3)) |
| 5 | Hep | - | - | Porcine intestinal mucosa, Sigma H3393 |
| 8 | CSA | 37,108 | 3,007 | Bovine trachea, Sigma C8529 |
| 9 | CSA (new) | 4,311 | 300 | Bovine trachea, Sigma C9819 |
| 10 | CSB | - | - | Bovine mucosa, Sigma C2413 |
| 13 | CSC | - | 18,406 | Shark cartilage, Sigma C4384 |
| 14 | CSC (new） | 21,162 | 34,541 | Shark cartilage, Sigma C4384 |

These analyses included two batches, each of CSA and CSC purchased from Sigma. They gave differing binding with the anti-KS MZ15. Binding scores are means of the fluorescence intensities at 0.1 ng/ spot; - indicates the score is less than 1. Relative binding intensity: <10%, 10-30%, 30-70%, 70-100%, where 100% is the maximum score for the respective antibodies.

**Table S2. Supplemental glycan microarray document based on MIRAGE Guidelines (doi:10.3762/mirage.3)**

| **Classification** | **Guidelines** |
| --- | --- |
| 1. **Sample: Glycan Binding Sample** | |
| Description of Sample | The antibodies, recombinant growth factors and morphogens, their sources, concentrations, and blocking conditions used in microarray analyses are summarized below.   \| Antibodies and proteins \| Isotypes \| Conc (μg/ml)  or dilutions \| Sources and references \| Blockers and diluents^a^ \| \| --- \| --- \| --- \| --- \| --- \| \| R-10G \| Mouse IgG \| 10 \| Millipore MABD151 \| Blocker B and Diluent 2 \| \| 100 \| Blocker B and Diluent 2 \| \| MZ15 \| Mouse IgG \| 10 \| ([Zanetti et al., J Cell Biol. 1985](https://www.ncbi.nlm.nih.gov/pubmed/?term=Two+subpopulations+of+differentiated+chondrocytes+identified+with+a+monoclonal+antibody+to+keratan+sulfate)) \| Blocker A and Diluent 1 \| \| 5D4 \| Mouse IgG \| 1/100 \| ([Caterson et al., J Biol Chem. 1983](https://www.ncbi.nlm.nih.gov/pubmed/?term=Identification+of+a+monoclonal+antibody+that+specifically+recognizes+corneal+and+skeletal+keratan+sulfate.+Monoclonal+antibodies+to+cartilage+proteoglycan.)) \| Blocker A and Diluent 1 \| \| CS56 \| Mouse IgM \| 1/200 \| Sigma C8035 \| Blocker A and Diluent 1 \| \| anti-i P1A ELL \| Human IgM \| 1/200 \| ([Gao et al., Anal Chem. 2015](https://www.ncbi.nlm.nih.gov/pubmed/?term=Negative-ion+electrospray+tandem+mass+spectrometry+and+microarray+analyses+of+developmentally+regulated+antigens+based+on+type+1+and+type+2+backbone+sequences.)) \| Blocker C and Diluent 3 \| \| Fc10.2 \| Mouse IgM \| Neat \| ([Gooi et al., Mol Immunol. 1983](https://www.ncbi.nlm.nih.gov/pubmed/?term=A+marker+of+human+foetal+endoderm+defined+by+a+monoclonal+antibody+involves+type+1+blood+group)) \| Blocker C and Diluent 3 \| \| TRA-1-60 \| Mouse IgM \| 50 \| Santa Cruz Biotechonology sc-21705 \| Blocker A and Diluent 1 \| \| TRA-1-81 \| Mouse IgM \| 50 \| Santa Cruz Biotechonology sc-21706 \| Blocker A and Diluent 1 \| \| Anti-LNT \| Mouse IgM \| 1/50 \| Signet Laboratories SIG-3310 \| Blocker D and Diluent 4 \| \| Recombinant human Wnt-3a \| - \| 10 \| R&D Systems 5036-WN \| Blocker A and Diluent 1 \| \| Recombinant human Shh \| - \| 10 \| Novusbio NBP2-35265 \| Blocker A and Diluent 1 \| \| Recombinant human FGF1 \| - \| 20 \| Sino Biological Inc.10013-HNAE \| Blocker A and Diluent 1 \| \| Recombinant human FGF2 \| - \| 20 \| Sino Biological Inc.10014-HNAE \| Blocker A and Diluent 1 \| \| Recombinant human BMP-2 \| - \| 10 \| R&D Systems 355-BM \| Blocker A and Diluent 1 \| \| Recombinant human BMP-4 \| - \| 10 \| R&D Systems 314-BP \| Blocker A and Diluent 1 \|   ^a^ The compositions of blockers and diluents are shown in ‘assay protocol’ below. |
| Sample modifications | Not relevant. |
| Assay protocol | Microarray analyses were performed essentially as described ([Liu et al., Methods Mol. Biol. 2012](https://www.ncbi.nlm.nih.gov/pubmed/22057521)), for modifications of the protocol please see Microarray analysis section under EXPERIMENTAL PROCEDURES.  The detection antibodies used and their source, concentrations used in the analyses are summarised below.   \| Detection antibodies \| Isotypes \| Conc (μg/ml) \| Sources and references \| Blockers and diluents^a^ \| \| --- \| --- \| --- \| --- \| --- \| \| Human/Mouse Wnt-3a antibody \| Rat IgG \| 2 \| R&D Systems MAB1324 \| Blocker A and Diluent 1 \| \| Human/Mouse Shh N-Terminus antibody \| Rat IgG \| 2 \| R&D Systems MAB464 \| Blocker A and Diluent 1 \| \| Human FGF1 antibody \| Mouse IgG \| 5 \| R&D Systems MAB232 \| Blocker A and Diluent 1 \| \| Human FGF2 antibody \| Mouse IgG \| 5 \| R&D Systems MAB233 \| Blocker A and Diluent 1 \| \| Human BMP-2/BMP-4 antibody \| Mouse IgG \| 2 \| R&D Systems MAB3552 \| Blocker A and Diluent 1 \| \| Goat anti-mouse IgG^b^ \| Polyclonal \| 2 \| Sigma B7264 \| Blocker A or B and Diluent 1 or 2^c^ \| \| Goat anti-mouse IgM^b^ \| Polyclonal \| 5 \| Sigma B9265 \| Blocker A and Diluent 1 \| \| Goat anti-human IgM^b^ \| Polyclonal \| 2.5 \| Vector BA-3020 \| Blocker C and Diluent 3 \| \| Goat anti-rat IgG^b^ \| Polyclonal \| 5 \| Vector BA-4000 \| Blocker A and Diluent 1 \|   ^a^ The compositions of blockers and diluents are shown in ‘assay protocol’ below.  ^b^ These are biotinylated antibodies.  ^c^ Blocker and Diluent were selected according to the corresponding primary antibody.   \| Blockers and diluents used are shown below. \| A \| 3% (w/v) bovine serum albumin (BSA) in phosphate buffered solution (PBS) \| \| --- \| --- \| --- \| \| B \| 1% (w/v) BSA containing 0.33% (w/v) casein in PBS \| \| C \| 3% (w/v) BSA and 5 mM CaCl_2_ in hepes-buffered saline (HBS) \| \| D \| 1% (w/v) BSA containing 0.02% (w/v) casein and 5 mM CaCl_2_ in HBS \| \| Diluents \| 1 \| 1% (w/v) BSA in PBS \| \| 2 \| 1% (w/v) BSA containing 0.33% (w/v) casein in PBS \| \| 3 \| 3% (w/v) BSA and 5 mM CaCl_2_ in HBS \| \| 4 \| 1% (w/v) BSA containing 0.02% (w/v) casein and 5 mM CaCl_2_ in HBS \| |
| **2.** **Glycan Library** | |
| Glycan description for defined glycans | Microarray of sequence-defined glycans (in house designation ‘CLL array’) contained 79 lipid-linked glycans, neoglycolipids (NGLs) and glycolipids. The probe names and structures are in Table S3.  Five KS derived 6 mers whose sequences were fully or partially determined (described in the RESULTS section) are listed below.   \| Designations of glycans \| Sequences \| \| --- \| --- \| \| K’ase II-6(3S) \| Galβ-4GlcNAcβ-3Galβ-4GlcNAcβ-3Galβ-4GlcNAc  │ │ │  6S 6S 6S \| \| K’ase II-6(2S)AC \| Galβ-4GlcNAcβ-3Galβ-4GlcNAcβ-3Galβ-4GlcNAc  │ │  6S 6S \| \| K’ase II-6(2S)BC \| Galβ-4GlcNAcβ-3Galβ-4GlcNAcβ-3Galβ-4GlcNAc  │ │  6S 6S \| \| K’ase II-6(1S)Cα \| Galβ-4GlcNAcβ-3Galβ-4GlcNAcβ-3Galβ-4GlcNAc  │  6S \| \| K’ase II-6(1S)Cβ \| Galβ-4GlcNAcβ-3Galβ-4GlcNAcβ-3Galβ-4GlcNAc  │  6S \|   Four glycans obtained after desulfation of K’ase II-6(4S) and K’ase II-6(5S), as described in the RESULTS section are listed below.   \| Designations \| Sequences \| \| --- \| --- \| \| K’ase II-6_d_(0S) \| Galβ-4GlcNAcβ-3Galβ-4GlcNAcβ-3Galβ-4GlcNAc \| \| K’ase II-6_d_(1S)A \| Galβ-4GlcNAcβ-3Galβ-4GlcNAcβ-3Galβ-4GlcNAc  │  6S \| \| K’ase II-6_d_(1S)B \| Galβ-4GlcNAcβ-3Galβ-4GlcNAcβ-3Galβ-4GlcNAc  │  6S \| \| K’ase II-6_d_(1S)C \| Galβ-4GlcNAcβ-3Galβ-4GlcNAcβ-3Galβ-4GlcNAc  │  6S \|   Five chemically synthesized glycans by TCI are listed below.   \| Designations \| Sequences \| Sources or refs \| \| --- \| --- \| --- \| \| TCI-4(0S) \| Galβ-4GlcNAcβ-3Galβ-4GlcNAc \| ([Hiromi et al., Glycoconj J. 2017](https://link.springer.com/article/10.1007/s10719-017-9765-8)) \| \| TCI-4(4S) \| Galβ-4GlcNAcβ-3Galβ-4GlcNAc  │ │ │ │  6S 6S 6S 6S \| \| TCI-6(1S)A \| Galβ-4GlcNAcβ-3Galβ-4GlcNAcβ-3Galβ-4GlcNAc  │  6S \| TCI L2-L1-L1 [L0340] \| \| TCI-6(2S)AB \| Galβ-4GlcNAcβ-3Galβ-4GlcNAcβ-3Galβ-4GlcNAc  │ │  6S 6S \| TCI L2-L2-L1 [L0343] \| \| TCI-6(2S)AC \| Galβ-4GlcNAcβ-3Galβ-4GlcNAcβ-3Galβ-4GlcNAc  │ │  6S 6S \| TCI L2-L1-L2 [L0342] \|   NGLs of the above-mentioned five KS-derived and five synthetic glycans as well as those of five sequence-defined glycans listed below ([Gao et al., Anal Chem. 2015](https://www.ncbi.nlm.nih.gov/pubmed/26530895); [Li et al., Mol Cell Proteomics. 2018](https://www.ncbi.nlm.nih.gov/pubmed/?term=O-Glycome+Beam+Search+Arrays+for+Carbohydrate+Ligand+Discovery)) were included in the primary, secondary, tertiary and quaternary Beam Search arrays described in the RESULTS section (in house designations KS OS sets 1, 2a, 2b and 3c).   \| Glycan name \| Sequences \| \| --- \| --- \| \| LNT \| Galβ-3GlcNAcβ-3Galβ-4Glc \| \| LNnT \| Galβ-4GlcNAcβ-3Galβ-4Glc \| \| pLNH \| Galβ-3GlcNAcβ-3Galβ-4GlcNAcβ-3Galβ-4Glc \| \| pLNnH \| Galβ-4GlcNAcβ-3Galβ-4GlcNAcβ-3Galβ-4Glc \| \| pLNH-b \| Galβ-3GlcNAcβ-3Galβ-3GlcNAcβ-3Galβ-4Glc \| |
| Glycan description for undefined glycans | Polysaccharide microarray (in house designation GAG polysaccharide microarray set 2) contained 12 polysaccharides, the origins and sources of which are described below.   \| Polysaccharides \| Abbreviations \| Origins \| Sources \| \| --- \| --- \| --- \| --- \| \| Hyaluronic acid \| HA \| Bovine vitreous humor \| Sigma H7630 \| \| Keratan sulfate \| KS (bc) \| Bovine cornea \| ([Weyers et al., FEBS J. 2013](https://www.ncbi.nlm.nih.gov/pubmed/?term=Isolation+of+bovine+corneal+keratan+sulfate+and+its+growth+factor+and+morphogen+binding)) \| \| Keratan sulfate \| KS (bac) \| Bovine articular cartilage \| ([Brown et al., Biochemistry. 1994](https://www.ncbi.nlm.nih.gov/pubmed/?term=Oligosaccharides+derived+from+bovine+articular+cartilage+keratan+sulfates+after+keratanase+II+digestion%3A+implications+for+keratan+sulfate+structural+fingerprintin)) \| \| Keratan sulfate \| KS (cew) \| Chicken egg white \| ([Fu et al., Glycobiology. 2016](https://www.ncbi.nlm.nih.gov/pubmed/26903438)) \| \| Chondroitin sulfate A \| CSA \| Bovine trachea \| Sigma C8529 \| \| Chondroitin sulfate B \| CSB \| Bovine mucosa \| Sigma C3788 \| \| Chondroitin sulfate C \| CSC \| Shark cartilage \| Sigma C4384 \| \| Heparin \| HEP \| Porcine intestinal mucosa \| Sigma H3149 \| \| Heparan sulfate \| HS (pi) \| Porcine intestinal mucosa, Fraction I \| Celsus Laboratories HO-10697  ([Mulloy et al., Anal Chem. 2016](https://www.ncbi.nlm.nih.gov/pubmed/?term=Abnormally+high+content+of+free+glucosamine+residues+identified+in+a+preparation+of+commercially+available+porcine+intestinal+heparan+sulfate.)) \| \| Heparan sulfate \| HS (bk) \| Bovine kidney \| Sigma H7640 \| \| Dextran sulfate \| Dex-S \| Synthesized \| Sigma 42867 \| \| Dextran T2000 \| Dex \| Synthesized \| Pharmacosmos 551020009007 \|   Undefined glycans in KS glycome arrays include those from partial depolymerization with keratanase I, keratanase II and mild acid hydrolysis. Their preparation is described in EXPERIMENTAL PROCEDURES and SUPPLEMETNAL RESULTS, and their designations are in Figure 4. |
| Glycan modifications | NGLs in the CLL microarray had been prepared from reducing oligosaccharides either by reductive amination with the amino lipid, 1,2-dihexadecyl-*sn*-glycero-3-phosphoethanolamine [(DHPE) [(Chai et al., Methods Enzymol. 2003)](https://www.ncbi.nlm.nih.gov/pubmed/12968363)]; or by oxime ligation with aminooxy functionalized DHPE [(AOPE)]. The NGL OY contains the 6-linked fragment, -OCH_2_-CH_2_-, of core GalNAcol of an *O*-glycan branch after periodate oxidation and conjugation to *N*-aminoacetyl-*N*-(9-anthracenylmethyl)-1,2-dihexadecyl-*sn*-glycero-3-phosphoethanolamine, ADHP [(Gao et al., J Biol Chem. 2014)](https://www.ncbi.nlm.nih.gov/pubmed/24753245).  NGLs derived from the KS-related natural and synthetic glycans were prepared by oxime ligation using AOPE as described in EXPERIMENTAL PROCEDURES. |
| 1. **3.** **Printing Surface; e.g., Microarray Slide** | |
| Description of surface | Nitrocellulose-coated glass microarray slides. |
| Manufacturer | Whatman® FAST 16-pad Microarray Slides were used for CLL microarray Set  16-pad UniSart® 3D Microarray Slide from Sartorius (Goettingen, Germany) were used for GAG polysaccharide Set 1 and KS OS Sets 2a, 2b and 3c. |
| Custom preparation of surface | Not relevant. |
| Non-covalent Immobilization | NGLs and glycolipids were formulated as liposomes by adding carrier lipids, phosphatidylcholine (or 1,2-dihexanoyl-*sn*-glycero-3-phosphocholine, designated as DHPC) and cholesterol ([Liu et al., Methods Mol. Biol. 2012](https://www.ncbi.nlm.nih.gov/pubmed/22057521)) for robotically arraying and non-covalent immobilization on nitrocellulose-coated glass slides.  Polysaccharides were immobilized non-covalently without any formulation.  Binding signals were glycan dose related. |
| **4. Arrayer (Printer)** | |
| Description of Arrayer | Nano-Plotter 2.1 (GeSim, Radeberg, Germany) |
| Dispensing mechanism | Non-contact liquid delivery with four dispensing tips. |
| Glycan deposition | Approximately 0.33 nl was printed per spot |
| Printing conditions | The printing solutions for polysaccharides, NGLs and glycolipids were all aqueous-based. Printing was performed at ambient temperature and relative humidity of 50-58%.  The printing solutions of polysaccharides were at 0.1 and 0.3 mg/ml for the 0.03 and 0.1 ng per spot levels, respectively.  The printing solutions for NGL arrays contained 100 pmol/μl each of cholesterol and phosphatidylcholine (or DHPC) as lipid carriers in addition to the lipid-linked glycan probes in water (HPLC grade). The concentrations of the lipid-linked glycan probes were 5 and 15 pmol/μl for the 2 and 5 fmol per spot levels, respectively.  All printing solutions contained Cy3 NHS ester (GE Healthcare) at 20 ng/ml (26 fmol/μl) as a marker to monitor the printing process. |
| 1. **5.** **Glycan Microarray with “Map”** | |
| Array layout | Each array slide contained 16 identical subarrays (pads). Each subarray contained up to 64 lipid-linked glycans or polysaccharides printed at the two levels in duplicate (four spots per saccharide in a row); up to 256 spots (16x16) in total for 64 probes. |
| Glycan identification and quality control | Quality control of the ‘GAG polysaccharide microarray set 1’ included analyses with mAbs MZ15, 5D4 and CS56 (Figure 1).  Quality control of the ‘CLL microarray set’ included analyses with anti-i P1A ELL and anti-LNT (Table S4), and analyses with anti-H type1, anti-H type 2, anti-A, anti-B, anti-SSEA-1, anti-Le^a^, anti-Le^b^,, anti-Le^x^, anti-Le^y^, anti-I Ma and anti-I Step, as well as plant lectins UEA-1, WGA, MAL-I (Table S4).  Quality control of the KS glycome arrays included analyses with anti-i P1A ELL and anti-KS mAb MZ15 (Figure 4). |
| 1. **6. Detector and Data Processing** | |
| Scanning hardware | GenePix 4300A (Molecular Devices, Berkshire, UK) |
| Scanner settings | Scanning resolution: 10 μm / pixel (this resolution is adequate for the sizes of sample spots)  Laser channel: Red (scan wavelength 635 nm)  PMT voltage: 350  Scan power: Adjusted for each sample to achieve maximum signal without saturation of any single spot. |
| Image analysis software | ScanArray Express software (PerkinElmer LAS, Beaconsfield, UK) for the CLL array and GenePix® Pro 7 (Molecular Devices, Berkshire, UK) for the polysaccharide and KS-related arrays. |
| Data processing | The gpr files were entered into an in-house microarray database using software (designed by Dr Mark Stoll, <http://www.beilstein-institut.de/en/publications/proceedings/glyco-2009>) for data processing. No particular normalization method or statistical analysis was used. |
| **7.** **Glycan Microarray Data Presentation** | |
| Data presentation | The microarray binding results are in Figures 1 and 4, Tables 2 and 3, as well as Tables S3, S4 and S7. Binding results are presented as fluorescence intensity of binding in scores and errors. |
| 1. **8.** **Interpretation and** **Conclusion from Microarray Data** | |
| Data interpretation | No software or algorithms were used to interpret processed data. |
| Conclusions | a) The glycan sequence recognized by mAb R-10G consists of the mono-sulfated type 2-type 2 chain, with sulfate at postion 6 of the non-reducing end *N*-acetylglucosamine which hinders recognition by anti-I PIA ELL.  b) The glycan sequences recognized by four other anti-human stem cell antibodies FC10.2, TRA-1-60, TRA-1-81 and anti-i PIA ELL are non-sulfated.  c) The mAbs FC10.2, TRA-1-60 and TRA-1-81 recognize the type 1-type 2-type 2 sequence  d) The mAb TRA-1-60 can accommodate the type 1-type 2-type 2 sequence with fucose at the inner *N*-acetylglucosamine   |

**Table S3. Sequences of glycan probes in CLL microarray**

| Probe position | Probe name | Structure |
| --- | --- | --- |
| 1 | Orsay-1 | ^^Galβ-4GlcNAcβ-6Gal-AO^a^ |
| 2 | Orsay-2 | Galβ-4GlcNAcβ-3Gal-AO |
| 3 | Orsay-3 | Galβ-3GlcNAcβ-6Gal-AO |
| 4 | Orsay-4 | Galβ-3GlcNAcβ-3Gal-AO |
| 5 | LNT | ^^Galβ-3GlcNAcβ-3Galβ-4Glc-DH^a^ |
| 6 | LNnT | Galβ-4GlcNAcβ-3Galβ-4Glc-DH |
| 7 | LSTa | NeuAcα-3Galβ-3GlcNAcβ-3Galβ-4Glc-DH |
| 8 | LSTc | NeuAcα-6Galβ-4GlcNAcβ-3Galβ-4Glc-DH |
| 9 | LSTd | NeuAcα-3Galβ-4GlcNAcβ-3Galβ-4Glc-DH |
| 10 | GSC-915-5 | GlcNAcβ-6Galβ-4GlcNAcβ-3Galβ-4Glc-DH |
| 11 | pLNH | Galβ-3GlcNAcβ-3Galβ-4GlcNAcβ-3Galβ-4Glc-DH |
| 12 | pLNnH | Galβ-4GlcNAcβ-3Galβ-4GlcNAcβ-3Galβ-4Glc-DH |
| 13 | GSC-915-4 (new) | Galβ-4GlcNAcβ-6Galβ-4GlcNAcβ-3Galβ-4Glc-DH |
| 14 | LNnO | Galβ-4GlcNAcβ-3Galβ-4GlcNAcβ-3Galβ-4GlcNAcβ-3Galβ-4Glc-DH |
| 15 | NeuAcα-(3')LNnO | NeuAcα-3Galβ-4GlcNAcβ-3Galβ-4GlcNAcβ-3Galβ-4GlcNAcβ-3Galβ-4Glc-DH |
| 16 | NeuAcα-(6')LNnO (F1)^b^ | NeuAcα-6Galβ-4GlcNAcβ-3Galβ-4GlcNAcβ-3Galβ-4GlcNAcβ-3Galβ-4Glc-DH |
| 17 | NeuAcα-(6')LNnO (F2)^b^ | NeuAcα-6Galβ-4GlcNAcβ-3Galβ-4GlcNAcβ-3Galβ-4GlcNAcβ-3Galβ-4Glc-DH |
| 18 | pHGGs |  |
| 19 | Orsay-5 |  GlcNAcβ-6  │  Gal-AO  │ Galβ-3GlcNAcβ-3 |
| 20 | Orsay-6 | Galβ-4GlcNAcβ-6  │  Gal-AO  │ Galβ-3GlcNAcβ-3 |
| 21 | Orsay-7 | Galβ-4GlcNAcβ-6  │  Gal-AO  │ Galβ-4GlcNAcβ-3 |
| 22 | LNH | Galβ-4GlcNAcβ-6  │  Galβ-4Glc-DH  │ Galβ-3GlcNAcβ-3 |
| 23 | LNnH | Galβ-4GlcNAcβ-6  │  Galβ-4Glc-DH  │ Galβ-4GlcNAcβ-3 |
| 24 | MSLNH | NeuAcα-6Galβ-4GlcNAcβ-6  │  Galβ-4Glc-DH  │  Galβ-3GlcNAcβ-3 |
| 25 | MSLNnH-I |  Galβ-4GlcNAcβ-6  │  Galβ-4Glc-DH  │ NeuAcα-6Galβ-3GlcNAcβ-3 |
| 26 | DSLNnH | NeuAcα-6Galβ-4GlcNAcβ-6  │  Galβ-4Glc-DH  │ NeuAcα-6Galβ-4GlcNAcβ-3 |
| 27 | iLNO | Galβ-3GlcNAcβ-3Galβ-4GlcNAcβ-6  │  Galβ-4Glc-DH   │  Galβ-3GlcNAcβ-3 |
| 28 | LND | Galβ-4GlcNAcβ-6  │  Galβ-4GlcNAcβ-6  │ │ Galβ-3GlcNAcβ-3 Galβ-4Glc-DH   │  Galβ-3GlcNAcβ-3 |
| 29 | Nonaosylceramide | ^^ GlcNAcβ-6  │ GlcNAcβ-6 Galβ-4GlcNAcβ-3Galβ-4Glcβ-Cer^a^  │ │  Galβ-4GlcNAcβ-3  │ GlcNAcβ-3 |
| 30 | I-octaosylceramide | Galβ-4GlcNAcβ-6  │  Galβ-4GlcNAcβ-3Galβ-4Glcβ-Cer  │  Galβ-4GlcNAcβ-3 |
| 31 | I-dodecaosylceramide |  Galβ-4GlcNAcβ-6  │ Galβ-4GlcNAcβ-6 Galβ-4GlcNAcβ-3Galβ-4Glcβ-Cer  │ │  Galβ-4GlcNAcβ-3  │ Galβ-4GlcNAcβ-3 |
| 32 | B-like decaosylceramide | Galα-3Galβ-4GlcNAcβ-6  │  Galβ-4GlcNAcβ-3Galβ-4Glcβ-Cer  │ Galα-3Galβ-4GlcNAcβ-3 |
| 33 | B-like pentadecaosylceramide |  Galα-3Galβ-4GlcNAcβ-6  │ Galα-3Galβ-4GlcNAcβ-6 Galβ-4GlcNAcβ-3Galβ-4Glcβ-Cer  │ │  Galβ-4GlcNAcβ-3  │ Galα-3Galβ-4GlcNAcβ-3 |
| 34 | LNFP-II | Galβ-3GlcNAcβ-3Galβ-4Glc-DH  │  Fucα-4 |
| 35 | LNDFH-I | Fucα-2Galβ-3GlcNAcβ-3Galβ-4Glc-DH  │  Fucα-4 |
| 36 | LNFP-III | Galβ-4GlcNAcβ-3Galβ-4Glc-DH  │  Fucα-3 |
| 37 | LNnDFH-I | Fucα-2Galβ-4GlcNAcβ-3Galβ-4Glc-DH  │  Fucα-3 |
| 38 | DFLNH(b) | Galβ-4GlcNAcβ-6  │ │  Fucα-3 Galβ-4Glc-DH  │  Galβ-3GlcNAcβ-3  │  Fucα-4 |
| 39 | TFLNH |  Galβ-4GlcNAcβ-6   │ │   Fucα-3 Galβ-4Glc-DH  │  Fucα-2Galβ-3GlcNAcβ-3   │   Fucα-4 |
| 40 | DFLNnH | Galβ-4GlcNAcβ-6  │ │  Fucα-3 Galβ-4Glc-DH  │ Galβ-4GlcNAcβ-3  │  Fucα-3 |
| 41 | MSDFLNnH |  Fucα-2Galβ-4GlcNAcβ-6   │ │  Fucα-3 Galβ-4Glc-AO  │  NeuAcα-6Galβ-4GlcNAcβ-3 |
| 42 | MFiLNO-IV | Galβ-3GlcNAcβ-3Galβ-4GlcNAcβ-6  │ │  Fucα-3 Galβ-4Glc-DH  │  Galβ-3GlcNAcβ-3 |
| 43 | DFiLNO | Galβ-3GlcNAcβ-3Galβ-4GlcNAcβ-6  │ │  Fucα-3 Galβ-4Glc-DH   │  Fucα-2Galβ-3GlcNAcβ-3 |
| 44 | TFiLNO(1-2,2,3) | Fucα-2Galβ-3GlcNAcβ-3Galβ-4GlcNAcβ-6  │ │  Fucα-3 Galβ-4Glc-DH   │  Fucα-2Galβ-3GlcNAcβ-3 |
| 45 | TFiLNO | Galβ-3GlcNAcβ-3Galβ-4GlcNAcβ-6  │ │ │  Fucα-4 Fucα-3 Galβ-4Glc-DH   │  Galβ-3GlcNAcβ-3  │  Fucα-4 |
| 46 | LNFP-I | Fucα-2Galβ-3GlcNAcβ-3Galβ-4Glc-DH |
| 47 | LnNFPI | Fucα-2Galβ-4GlcNAcβ-3Galβ-4Glc-DH |
| 48 | H2 (with H2+Fuc)* | Fucα-2Galβ-4GlcNAcβ-3Galβ-4GlcNAcβ-3Galβ-4Glcβ-Cer |
| 49 | Globo-H-Hexa | Fucα-2Galβ-3GalNAcβ-3Galα-4Galβ-4GlcNAc-DH |
| 50 | MFLNH-I |  Galβ-4GlcNAcβ-6  │  Galβ-4Glc-DH  │  Fucα-2Galβ-3GlcNAcβ-3 |
| 51 | PSM-F1H2HN3 | Fucα-2Galβ-4GlcNAcβ-6   │   Galβ-4GlcNAcβ-OY^a^   │   GlcNAcβ-3 |
| 52 | GSC-915-3 | Fucα-2Galβ-4GlcNAcβ-6Galβ-4GlcNAcβ-3Galβ-4Glc-DH |
| 53 | GSC-915-2 | Fucα-2Galβ-4GlcNAcβ-6   │   Galβ-4GlcNAcβ-3Galβ-4Glc-DH   │   GlcNAcβ-3 |
| 54 | GSC-915 | Fucα-2Galβ-4GlcNAcβ-6   │   Galβ-4GlcNAcβ-3Galβ-4Glc-DH   │   Galβ-4GlcNAcβ-3 |
| 55 | GSC-915-AO | Fucα-2Galβ-4GlcNAcβ-6   │   Galβ-4GlcNAcβ-3Galβ-4Glc-AO  │   Galβ-4GlcNAcβ-3 |
| 56 | H3 (with H3-Fuc)* | Fucα-2Galβ-4GlcNAcβ-6   │   Galβ-4GlcNAcβ-3Galβ-4Glc-Cer  │  Fucα-2Galβ-4GlcNAcβ-3 |
| 57 | A-Hexa-T1 | GalNAcα-3Galβ-3GlcNAcβ-3Galβ-4Glc-DH  │  Fucα-2 |
| 58 | A-Hexa-T2 | GalNAcα-3Galβ-4GlcNAcβ-3Galβ-4Glc-DH  │  Fucα-2 |
| 59 | Globo-A-Hepta | GalNAcα-3Galβ-3GalNAcβ-3Galα-4Galβ-4GlcNAc-DH  │  Fucα-2 |
| 60 | A-Hepta |  Fucα-4  │ GalNAcα-3Galβ-3GlcNAcβ-3Galβ-4Glc-DH  │  Fucα-2 |
| 61 | Ab (with Ab+Fuc)* | GalNAcα-3Galβ-4GlcNAcβ-3Galβ-4GlcNAcβ-3Galβ-4Glcβ-Cer  │  Fucα-2 |
| 62 | Ad (with Ad+Fuc)* |  GalNAcα-3Galβ-4GlcNAcβ-6  │ │  Fucα-2 Galβ-4GlcNAcβ-3Galβ-4Glcβ-Cer  │ GalNAcα-3Galβ-4GlcNAcβ-3Galβ-4GlcNAcβ-3  │   Fucα-2 |
| 63 | B-Hexa-T1 | Galα-3Galβ-3GlcNAcβ-3Galβ-4Glc-DH  │  Fucα-2 |
| 64 | B-Hexa-T2 | Galα-3Galβ-4GlcNAcβ-3Galβ-4Glc-DH  │  Fucα-2 |
| 65 | B-penta | Galα-3Galβ-4Glc-AO  │ │  Fucα-2 │  Fucα-3 |
| 66 | Globo-B-Hepta | Galα-3Galβ-3GalNAcβ-3Galα-4Galβ-4GlcNAc-DH  │  Fucα-2 |
| 67 | B-III dodecaosylceramide | Galα-3Galβ-4GlcNAcβ-6  │ │  Fucα-2 Galβ-4GlcNAcβ-3Galβ-4Glcβ-Cer  │ Galα-3Galβ-4GlcNAcβ-3  │  Fucα-2 |
| 68 | B-IV tetradecaosylceramide |  Galα-3Galβ-4GlcNAcβ-6  │ │  Fucα-2 Galβ-4GlcNAcβ-3Galβ-4Glcβ-Cer  │ Galα-3Galβ-4GlcNAcβ-3Galβ-4GlcNAcβ-3  │  Fucα-2 |
| 69 | O1 | GlcNAcβ-6  │  Gal-AO  │ GlcNAcβ-3 |
| 70 | GalNAcα-3Galβ-4Glc | GalNAcα-3Galβ-4Glc-DH |
| 71 | Galα-4Galβ-4GlcNAc | Galα-4Galβ-4GlcNAc-DH |
| 72 | Globotri | Galα-4Galβ-4Glc-AO |
| 73 | Globoside (P-antigen) | GalNAcβ-3Galα-4Galβ-4Glcβ-Cer |
| 74 | B-like pentaosylceramide | Galα-3Galβ-4GlcNAcβ-3Galβ-4Glcβ-Cer |
| 75 | Klaus glycolipid | Galβ-3Galβ-4GlcNAcβ-3Galβ-4Glcβ-Cer |
| 76 | Forssmann glycolipid | GalNAcα-3GalNAcβ-3Galα-4Galβ-4Glcβ-Cer |
| 77 | SA4(α8) | NeuAcα-8NeuAcα-8NeuAcα-8NeuAc-DH |
| 78 | Lam-4 | Glcβ-3Glcβ-3Glcβ-3Glc-AO |
| 79 | Pust-4 | Glcβ-6Glcβ-6Glcβ-6Glc-AO |

^a^ AO, DH and OY are different lipid tags of NGLs as explained in MIRAGE document Supplemental Table S5; Cer is ceramide.

^b^ F1 and F2 are NGLs derived from different fractions of experimentally generated sialyl glycan products using 6’sialyl transferase.

Asterisked are glycolipid preparations that contain a minor component with an additional fucose residue detected by MALDI-MS; most likely these are part of the difucosylated Le^y^ sequence.

Table S4. Results of binding of mAbs R-10G, anti-i P1A ELL, FC10.2, TRA-1-60, TRA-1-81 and anti-LNT to glycan probes in CLL glycan microarray set.

The binding scores are means of the fluorescence intensity at 5 fmol/probe spot; errors represent half of the difference of signal intensities of duplicate spots for each glycan probe. - indicates the score less than 1. Relative binding intensity: <10%, 10-30%, 30-70%, 70-100%, where 100% is the maxim binding score for the reactive antibodies. N/A, array data not available due to the absence of the probes in the array.

**Table S5. Quality control data for CLL glycan microarray set: results of binding of selected plant lectins and anti-carbohydrate antibodies.**

The binding scores are means of the fluorescence intensity at 5 fmol/probe spot; errors represent half of the difference of signal intensities of duplicate spots for each glycan probe. - indicates the score less than 1. Relative binding intensity: <10%, 10-30%, 30-70%, 70-100%, where 100% is the maxim binding score for the reactive antibodies.

Abbreviations and sources of the proteins: AAL, Biotinylated Aleuria Aurantia Lectin (Vector Laboratories, B-1395); UEA-I, Biotinylated Ulex Europaeus Agglutinin I (Vector Laboratories, B-1065); anti-H type1, blood group H1 (O) antigen antibody [17-206] (Abcam, ab3355); anti-H type 2, anti-ABO antibody [BRIC231] (Abcam, ab33404); anti-A, blood group A antigen antibody (Abcam, ab3353); anti-B, blood group B antigen antibody (89-F) (Santa Cruz, sc-52371); anti-Le^a^, Lewis A antibody (Signet Laboratories, SIG-3314); anti-Le^b^, Lewis B antibody (Signet Laboratories, SIG-3315); anti-Le^x^, Lewis X antibody (Signet Laboratories, SIG-3339); anti-SSEA-1 ([Gooi et al., 1981](#_ENREF_2)); anti-Le^y^, Lewis Y antibody (Signet Laboratories, SIG-3317); RCA-I, Biotinylated Ricinus Communis Agglutinin I (Vector Laboratories, B-1085); anti-I Ma and ant-I Step ([Feizi 1981](#_ENREF_1)); WGA, Biotinylated Wheat Germ Agglutinin (Vector Laboratories, B-1025); MAL-I, Biotinylated Maackia Amurensis Lectin I (Vector Laboratories, B-1315).

**Table S6. Negative-ion ESI-MS analysis of glycans up to heptasaccharides obtained after size fractionations of bovine corneal KS following keratanase I or keratanase II partial digestions or mild acid treatment.**

| Depolymerization method | Fractions^a^ | Glycans | |
| --- | --- | --- | --- |
|  |  | Detected ions (*m/z*)^b^ | Deduced compositions^c^ |
| Keratanase I | F1 | 462 (z=1) | Gal1.GlcNAc1.S1 |
|  | F2 | 453.1 (z=2)  504.1 (z=2)  328.4 (z=2)  827.2 (z=1) | Gal2.GlcNAc2.S2  Gal2.GlcNAc2.S3.Na1  Gal2.GlcNAc2.S3  Gal2.GlcNAc2.S1 |
|  | F3 | 635.7 (z=2)  450.1 (z=3)  686.6 (z=2)  734.6 (z=2)  785.6 (z=2) | Gal3.GlcNAc3.S2  Gal3.GlcNAc3.S3  Gal3.GlcNAc3.S3.Na1  Gal3.GlcNAc3.S4.K  Gal3.GlcNAc3.S5.Na1.K1 |
| Keratanase II | F1 | 419.6 (z=3)  629.2 (z=2)  640.9 (z=2) | Gal1.GlcNAc1.S2  Gal1.GlcNAc1.S2  Gal1.GlcNAc1.S2.Na1 |
|  | F2 | 328.4 (z=3)  504.1 (z=2)  266 (z=4)  453.1 (z=2) | Gal2.GlcNAc2.S3  Gal2.GlcNAc2.S3.Na1  Gal2.GlcNAc2.S4  Gal2.GlcNAc2.S2 |
|  | F3 | 450.1 (z=3)  686.7 (z=2)  357.3 (z=4)  484.1 (z=3)  737.7 (z=2)  635.7 (z=2) | Gal3.GlcNAc3.S3  Gal3.GlcNAc3.S3.Na1  Gal3.GlcNAc3.S4  Gal3.GlcNAc3.S4.Na1  Gal3.GlcNAc3.S4.Na2  Gal3.GlcNAc3.S2 |
| Mild acid | F1 | 462 (z=1)  564 (z=1)  624 (z=1)  665 (z=1)  767 (z=1)  865 (z=1)  351 (z=2)  827 (z=1)  929 (z=1) | Gal1.GlcNAc1.S1  Gal1.GlcNAc1.S2.Na1  Gal2.GlcNAc1.S1  Gal1.GlcNAc2.S21  Gal1.GlcNAc2.S2  Gal2.GlcNAc1.S3.Na1. H_2_O  Gal2.GlcNAc1.S2  Gal2.GlcNAc2.S1  Gal2.GlcNAc2.S2.Na1 |
|  | F2 | 827 (z=1)  453.1 (z=2)  929 (z=1)  534.2 (z=2)  382.4 (z=3)  396.1 (z=3)  635.7 (z=2)  716.7 (z=2)  767.7 (z=2)  504.2 (z=3)  397.9 (z=3)  736.7 (z=2)  788.2 (z=2) | Gal2.GlcNAc2.S1  Gal2.GlcNAc2.S2  Gal2.GlcNAc2.S2.Na1  Gal3.GlcNAc2.S2  Gal3.GlcNAc2.S3  Gal2.GlcNAc3.S3  Gal3.GlcNAc3.S2  Gal4.GlcNAc3.S2  Gal4.GlcNAc3.S3.Na1  Gal4.GlcNAc3.S3  Gal4.GlcNAc3.S4  Gal3.GlcNAc4.S2  Gal3.GlcNAc4.S3.Na1 |

^a^ Fractions with longer glycans gave complex spectra and are not shown

^b^*m/z* is mass to charge ratio.

^c^Gal, Galactose; GlcNAc, N-acetylglucosamine; S, sulphate. The numbers in the deduced compositions refer to monosaccharides, sulphates, sodium (Na) and potassium (K) per molecule of oligosaccharide.

Table S7. Negative-ion ESI-MS analysis of glycan subtractions obtained by SAX-HPLC of K’ase II-6 mer fraction and of the derived NGLs.

| Fractions | Oligosaccharides | | NGLs | | Assignment of major component^a^ |
| --- | --- | --- | --- | --- | --- |
|  | Detected ions (*m/z*) | Deduced compositions | Detected ions (*m/z*) | Deduced compositions |  |
| HPLC-1 | 595.6 (z=2)  1192.3 (z=1) | Gal3.GlcNAc3.S1 | 955.4 (z=2) | Gal3.GlcNAc3.S1.AO | K’ase II-6(1S) |
| HPLC-2 | 635.7 (z=2) | Gal3.GlcNAc3.S2 | 995.0 (z=2)  663.0 (z=3)  2012.9 (z=1) | Gal3.GlcNAc3.S2.AO  Gal3.GlcNAc3.S2.Na1.AO | K’ase II-6(2S) |
| HPLC-3 | 450.1 (z=3)  686.6 (z=2) | Gal3.GlcNAc3.S3  Gal3.GlcNAc3.S3.Na | 516.9 (z=4)  696.9 (z=3) | Gal3.GlcNAc3.S3.AO  Gal3.GlcNAc3.S3.Na1.AO | K’ase II-6(3S) |
| HPLC-4 | 357.4 (z=4)  450.2 (z=3)  484.2 (z=3 | Gal3.GlcNAc3.S4  Gal3.GlcNAc3.S3  Gal3.GlcNAc3.S4.Na1 | 429.3 (z=5)  542.4 (z=4)  536.9 (z=4) | Gal3.GlcNAc3.S4.AO  Gal3.GlcNAc3.S4.Na1.AO  Gal3.GlcNAc3.S4.AO | K’ase II-6(4S) |
| HPLC-5 | 357.4 (z=4)  450.2 (z=3)  484.2 (z=3) | Gal3.GlcNAc3.S4  Gal3.GlcNAc3.S3  Gal3.GlcNAc3.S4.Na1 | 429.3 (z=5)  542.4 (z=4)  536.9 (z=4) | Gal3.GlcNAc3.S4.AO  Gal3.GlcNAc3.S4.Na1.AO  Gal3.GlcNAc3.S4.AO | K’ase II-6(4S) |
| HPLC-6 | 301.7 (z=5)  357.4 (z=4)  382.9 (z=4) | Gal3.GlcNAc3.S5  Gal3.GlcNAc3.S4  Gal3.GlcNAc3.S5.Na1 | 370.9 (z=6)  429.3 (z=5)  449.9 (z=5) | Gal3.GlcNAc3.S5.AO  Gal3.GlcNAc3.S4.AO  Gal3.GlcNAc3.S5.Na1.AO | K’ase II-6(5S) |
| HPLC-7 | 301.7 (z=5)  382.9 (z=4)  357.4 (z=4) | Gal3.GlcNAc3.S5  Gal3.GlcNAc3.S5.Na1  Gal3.GlcNAc3.S4 | 371.1 (z=6)  429.3 (z=5)  449.9 (z=5)  567.9 (z=4) | Gal3.GlcNAc3.S5.AO  Gal3.GlcNAc3.S4.AO  Gal3.GlcNAc3.S5.Na1.AO  Gal3.GlcNAc3.S5.Na2.AO | K’ase II-6(5S) |
| HPLC-7a | - | - | 425.7 (z=4)  356.3 (z=5)  445.6 (z=4)  370.9 (z=6) | Gal2.GlcNAc2.S3.AO  Gal2.GlcNAc2.S4.AO  Gal2.GlcNAc2.S4.AO  Gal3.GlcNAc3.S5.AO | K’ase II-4(3S)  K’ase II-4(4S) |
| HPLC-8 | - | - | 371.0 (z=6)  429.3 (z=5)  449.7 (z=5)  562.4 (z=4) | Gal3.GlcNAc3.S5.AO  Gal3.GlcNAc3.S4.AO  Gal3.GlcNAc3.S5.AO  Gal3.GlcNAc3.S5.AO | K’ase-II-6(5S) |
| HPLC-8a | - | - | 343.9 (z=6)  396.9 (z=5)  417.3 (z=5)  521.9 (z=4)  527.4 (z=4) | Gal2.GlcNAc3.S5.AO  Gal2.GlcNAc3.S4.AO  Gal2.GlcNAc3.S5.Na1.AO  Gal2.GlcNAc3.S5.Na1.AO  Gal2.GlcNAc3.S5.Na2.AO | K’ase II-5(5S) |
| HPLC-9 | - | - | 329.2 (z=2)  371.0 (z=6)  387.9 (z=6)  449.7 (z=5) | Gal3.GlcNAc3.S6.AO  Gal3.GlcNAc3.S5.AO  Gal3.GlcNAc3.S6.Na1.AO  Gal3.GlcNAc3.S5.Na1.AO | K’ase II-6(6S) |

^a^The numbers in brackets, 1S-6S refer to the number of sulfate residues
